# DNA barcodes for rapid, whole genome, single-molecule analyses

**DOI:** 10.1093/nar/gkz212

**Published:** 2019-03-28

**Authors:** Nathaniel O Wand, Darren A Smith, Andrew A Wilkinson, Ashleigh E Rushton, Stephen J W Busby, Iain B Styles, Robert K Neely

**Affiliations:** 1 School of Chemistry, University of Birmingham, Edgbaston, Birmingham B15 2TT, UK; 2 Physical Sciences of Imaging in the Biomedical Sciences Centre for Doctoral Training, University of Birmingham, Edgbaston, Birmingham B15 2TT, UK; 3 School of Biosciences, University of Birmingham, Edgbaston, Birmingham B15 2TT, UK; 4 School of Computer Science, University of Birmingham, Edgbaston, Birmingham B15 2TT, UK

## Abstract

We report an approach for visualizing DNA sequence and using these ‘DNA barcodes’ to search complex mixtures of genomic material for DNA molecules of interest. We demonstrate three applications of this methodology; identifying specific molecules of interest from a dataset containing gigabasepairs of genome; identification of a bacterium from such a dataset and, finally, by locating infecting virus molecules in a background of human genomic material. As a result of the dense fluorescent labelling of the DNA, individual barcodes of the order 40 kb pairs in length can be reliably identified. This means DNA can be prepared for imaging using standard handling and purification techniques. The recorded dataset provides stable physical and electronic records of the total genomic content of a sample that can be readily searched for a molecule or region of interest.

## INTRODUCTION

Direct visualization of the DNA sequence by optical mapping offers a unique perspective on genome structure (1). The single-molecule, long-range information that is derived from mapping has recently been invaluable in revealing hundreds of large genomic rearrangements of the human (2,3) and great ape (4) genomes. However, the development of nanopore sequencing has enabled sequence read lengths that are of the same scale as optical maps (5), thereby providing an alternative approach for scaffolding or assembling sequencing data from a dataset which is far more information rich than the comparative mapping data.

Whilst nanopore sequencing is increasing the competition in the space traditionally occupied by optical mapping, the commercial Bionano Genomics platform continues to offer a valuable and reliable means of deriving a reference scaffold for genomic assemblies (6). Furthermore, imaging is particularly suited to multiplexed experiments, meaning that mapping offers a route by which whole genome studies can be performed, where sequence data can be directly correlated to, for example, a DNA repair event (7), DNA replication (8), or protein binding (9), at the single-molecule level. Whilst the promise of single-molecule mapping approaches for imaging DNA-based events has been demonstrated in such experiments, none to date has approached achieving this on the scale of a whole bacterial or human genome.

Several approaches exist for producing optical maps of DNA sequence (10). Until recently, mapping has been reliant on either restriction enzymes (11) or nicking enzymes (2) to define the sequence motifs used in mapping. However, the discovery that methyltransferase enzymes can be used for fluorescent labelling of DNA (9,12–14) has been transformative because of their ability to yield sequence-specifically modified DNA without introducing damage (cuts or nicks) to the DNA. Previous mapping approaches have focussed on mapping of long DNA molecules, typically greater than 200 kb in length, driven by the relative infrequency of mapping sites. Whether defined by restriction, nicking or methyltransferase enzyme, a map must carry enough information for it to be reliably matched to a sequence of interest, and hence, applied. For a 5- or 6-base targeting enzyme, map density is typically one site every 10 kb, resulting in the need for molecules hundreds of kb in length for reliable map assembly. The production and handling of DNA molecules on this size range is technically challenging, requiring careful extraction and clean-up of DNA using gels to minimize shearing forces in the sample (15).

We describe a methodology that significantly increases the accessibility and range of potential applications for optical mapping. We show that a high-density, yet sequence-specific labelling pattern, directed by a DNA methyltransferase allows single DNA molecules to be probed for sequences of interest at the whole-genome scale. Map analysis is demonstrated using relatively small DNA fragments (∼30 kb) that can be readily prepared using standard DNA extraction and purification kits and protocols. We show how this can be used to build datasets of hundreds of thousands of DNA molecules (several gigabasepairs of DNA) in less than an hour using standard fluorescence microscopy. To extend the imaging to applications in genomics, we have developed image-matching and classification techniques, which enable unique, whole genome analyses to be performed. For example, hundreds-of-thousands of single-molecule barcodes can be queried for a sequence of interest, and barcode images can be clustered, based on similarity, allowing the prevalent DNA molecules in a mixed population to be identified. We demonstrate application to the detection of viral infection in human cells, to identification of bacteria, and to the visualization of a region of interest in a genome that had been modified using CRISPR–Cas9 genome editing. In all, we expect this approach to find widespread application in the quantitative study of mixed genome samples and, particularly in studying the sequence context of genome-wide events and processes, such as replication or protein binding, that are not directly accessible with sequencing technologies.

## MATERIALS AND METHODS

### Labelling of genomic DNA

A 200 μl solution containing 1× CutSmart Buffer (NEB), 10 μg genomic DNA, 0.9 μg TaqI DNA methyltransferase (M.TaqI) and 750 μM AdoHcy-azide (Supplementary Figure S1) was prepared and incubated at 50°C for 1 h. Subsequently, 5μl 18mg/ml proteinase K (NEB)/0.1% Triton X-100 (Sigma-Aldrich) was added and this was incubated at 50°C for 1 h, before purification by GenElute Bacterial Genomic DNA kit (Sigma-Aldrich). DNA was eluted into 200 μl TE Buffer (10 mM tris, 1 mM EDTA). Meanwhile, a 20 μl solution containing 0.5× phosphate buffered saline (Sigma-Aldrich), 10 μl DMSO, 1 mM dibenzylcyclooctyne-amine (Sigma-Aldrich) and 12.5 mM Atto 647N-NHS ester (Sigma-Aldrich) was incubated at 4°C for 1 h. The DNA sample was split into 30 μl aliquots and 10 μl of the mixture containing the Atto 647N was added to an aliquot. This mixture was incubated at room temperature overnight, before purification by GenElute Bacterial Genomic DNA kit and eluted into 50 μl TE buffer (10 mM tris, 1 mM EDTA).

### Molecular combing

Molecular combing of DNA was performed based on the procedure described by Deen *et al.* (16). Glass coverslips (Borosilicate Glass No. 1, Thermo Fisher) were cleaned to remove any fluorescent contaminants by incubation in a furnace oven at 450°C for 24 h. After removing from the furnace and allowing to cool, 30 μl of Zeonex solution (Zeon Chemicals, 1.5% w/v solution Zeonex 330R in chlorobenzene) was deposited onto a coverslip on a spin coater (Ossila) and subsequently spun at 3000 rpm for 90 seconds. Zeonex-coated coverslips were allowed to dry at room temperature overnight and stored in a desiccator.

To perform the molecular combing, 2 μl Atto 647N-labelled DNA (2 ng/μl in 1× TE) was suspended in 17 μl 100 mM sodium phosphate buffer (pH 5.7) containing 1 μl DMSO. A 1.5 μl droplet of this solution was deposited on the surface of the Zeonex-coated coverslip. A clean pipette tip was placed in contact with the droplet and used to drag it, with a velocity of approximately 5 mm/min, across the coverslip.

### Fluorescence microscopy

Deposited DNA was imaged on an ASI RAMM microscope, equipped with a Nikon 100× TIRF objective. Illumination was from a 100 mW OBIS 640 nm CW laser via a quad-band dichroic mirror (405/488/561/635) and images were collected using an Evolve Delta EM-CCD camera, via a quad-band emission filter (Semrock, 432/515/595/730 nm). Micromanager was used to control the system and scan the sample (17).

### DNA barcode extraction, pairwise alignment and community detection

**Figure 1.**
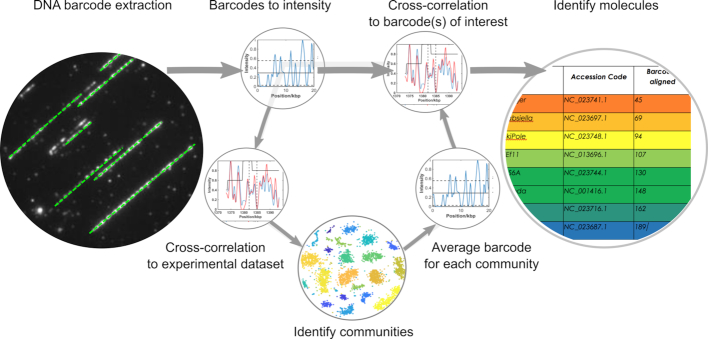
Overview of the computational steps taken to extract DNA barcodes from images and match them to a genome. Intensity profiles for each extracted barcode can either be directly compared to a database of molecules of interest or to every other barcode in the experimental dataset. In the latter approach, communities of similar DNA molecules can be identified and an average barcode for each community determined. Hence a single barcode for each community can be matched to a large database of possible genomes.

Software was written in MATLAB (R2016b, The MathWorks, Inc., Natick, MA, USA) for the automated extraction of DNA barcodes from microscopy images, in silico generation of DNA barcodes and the alignment procedures. The computational process is outlined in Figure 1. Full details of the procedure for these processes are given in the Supplementary Information and the code we used to extract, process and analyse DNA barcodes are available at edata.bham.ac.uk, DOI: 10.25500/eData.bham.00000255.

## RESULTS AND DISCUSSION

We label DNA using the M.TaqI DNA methyltransferase enzyme to direct the conjugation of fluorophores to sites reading 5′-TCGA-3′ (one site every 256 bp, on average). The M.TaqI enzyme is well-suited to DNA labelling using synthetically-prepared analogues of *S*-adenosyl-l-methionine and we use it here to add reactive azide groups to target adenine bases. We tested a range of conditions for labelling and several DBCO-conjugated organic dyes and developed conditions that give approximately nine fluorophores for every 10 labelling sites on long, genomic DNA molecules (14,18). Labelled DNA was separated from the methyltransferase enzyme and reactive dye using a standard silica-based column (for genome purification).

In a typical experiment, we deposit hundreds of gigabases of DNA barcodes from a 1 μl droplet containing 100 pg of DNA (16). A fraction of this, typically a few gigabases, is imaged for analysis. We visualize of the order of 10 000 molecules in 1000 fields of view in ∼20 min of imaging (Supplementary Figure S2 shows an example dataset). Software for the automated extraction of the images of DNA molecules from these datasets was developed and is described in the Supporting Information and in Supplementary Figure S3. Each DNA barcode extracted from the imaging data is stored as a string of integers, where each integer represents the intensity of an individual pixel along the DNA image. Initially, this dataset is filtered to remove molecules that are entwined/aggregated (using an average intensity threshold) and those that are estimated to be shorter than 30 kb in length.

**Figure 2.**
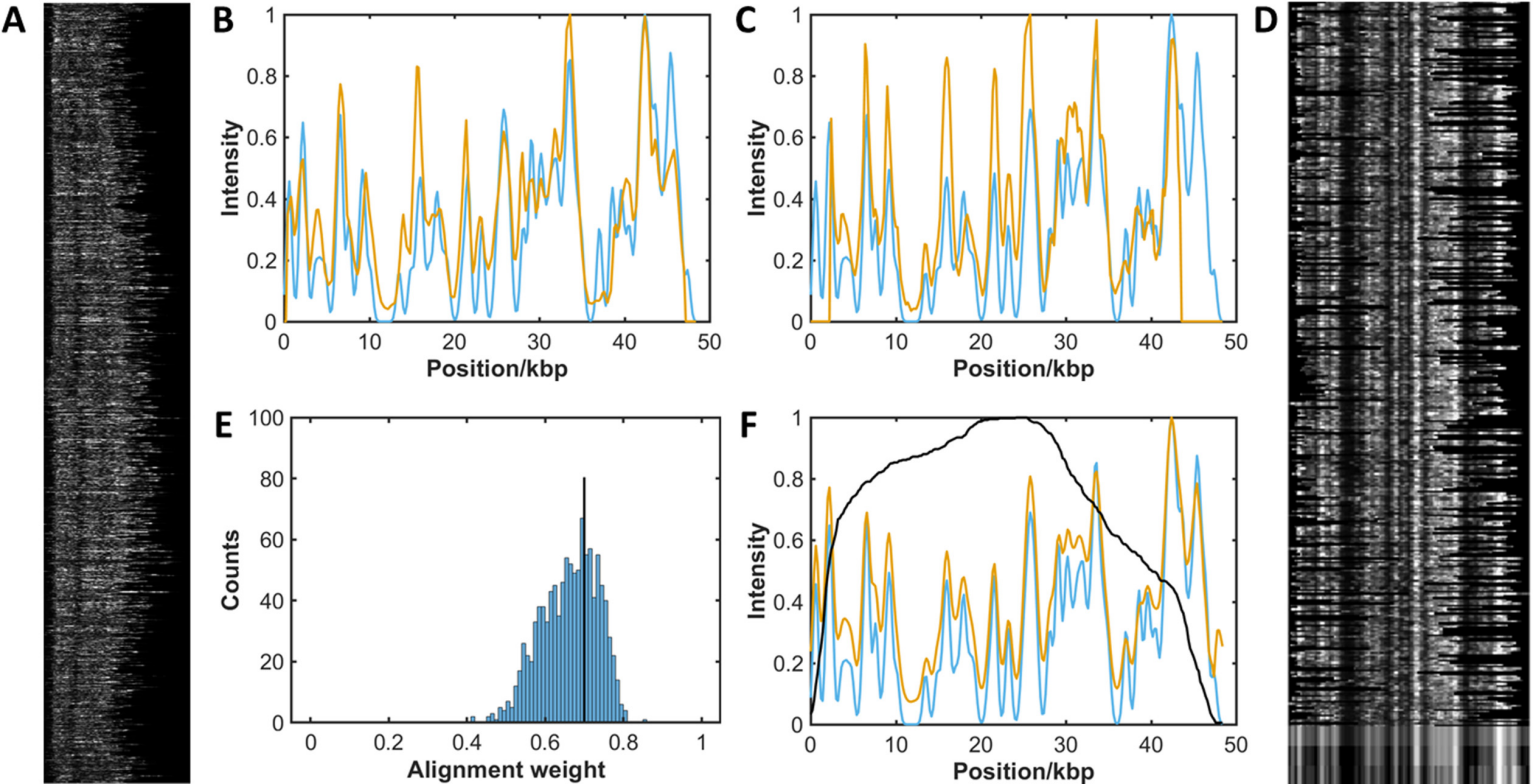
Alignment of pure experimental sample of the bacteriophage lambda genome. DNA was labelled with Atto647N using M.TaqI to direct labelling, combed and imaged. 38,000 candidate DNA barcodes were extracted from the images. (**A**) After filtering the experimental data, 1077 barcodes are identified for further analysis. (**B** and **C**) The fluorescence intensity profile of each experimental DNA barcode (red) is cross-correlated with a reference molecule (blue) to find the optimal stretch and alignment of the data. (**D**) Selected barcodes (368) with an alignment weight greater than the threshold. At the bottom of the image is shown the mean experimental barcode, the mean with the background removed and the reference barcode (top to bottom). (**E**) Alignment weight for all experimental barcodes is calculated and a threshold (of 0.7 in this case) is applied (black line). (**F**) Plot of mean experimental barcode (red) against the reference barcode (blue), with the number of barcodes (black).

An overview of the matching process for a dataset is given in Figure 1 and shown for experimental data in Figure 2. The process of matching the intensity profiles of two barcodes (experiment/reference or experiment/experiment) is a two-step procedure (see SI for a more detailed description):

1. Find the optimal cross correlation between the two barcodes. Both the relative displacement of the two barcodes and the stretch of the experimental barcode are optimized.
2. Determine the alignment weight.

All of the deposited DNA molecules are (over)stretched on the slide during combing. This is due to the force, generated as the DNA leaves the droplet, which gives a consistent stretch of the DNA to around 1.6 times that of B-form DNA, i.e. the step between base pairs is ∼0.54 nm on the surface, on average. We correct for this in the alignment to a reference molecule and allow the experimental barcode to vary in length by ±10%, to account for any inconsistency in the stretch.

We apply two general approaches for interrogation of a dataset of DNA barcodes, each revealing complementary information on the composition of the sample:

1. Pairwise matching: Direct matching of experimental barcodes to a reference library
2. A similarity search: Matching the dataset to itself in search of communities of similar molecules. Subsequent matching of average barcodes from communities to a reference library.

In order to develop an understanding of the scope of the mapping data, and the questions we might address using it, initially we examined each of these analytical approaches using data generated *in silico*.

### An *in silico* assessment of the impact of experimental variables on mapping

DNA barcodes were generated as strings of integers *in silico* and used in Monte Carlo simulations to understand the impact of a range of experimental variables on our ability to match a DNA barcode to a given DNA sequence. Barcodes were generated from the *Escherichia coli* K-12 genome sequence using the parameters described in Supplementary Table S1. A summary of the results from these simulations is given in the Supplementary Figures S4 and S5. We find that, for example, for DNA molecules >30 kb in length, a labelling efficiency of one fluorophore per palindromic target site will allow accurate matching of 90% of the experimental data to the 4.6 Mb *E. coli* genome, Supplementary Figure S5. As a result, this length threshold has been applied to the analyses presented, henceforth. The ability to match such short DNA barcodes to a reference genome highlights a significant advantage of using a high density of DNA labelling for the mapping experiment. The preparation of ultra-long DNA molecules is time-consuming and requires significant expertise, yet molecules 30–50 kb in length can be readily prepared using standard sample preparation kits for genomic DNA extraction and purification. As well as DNA labelling efficiency, and DNA length, we find that non-specific labelling and the signal-to-noise ratio in the imaging are important parameters for generating reliable fits but factors such as a variation in fluorophore intensity, the degree of stretching of the DNA barcode and image resolution have little impact on matching, within the bounds we tested.

We also used the barcodes we generated *in silico* to improve our measure of the goodness of fit between a barcode and its reference sequence. These calculations show that an approach relying solely on the normalized cross-correlation between barcodes does not give a sufficiently discriminating measure of the goodness of fit to resolve correctly- and incorrectly-fitted populations of molecules (for the simulated sample of *E. coli* K-12 genome). In order to address this, we introduced an alignment weighting, which is calculated as the mean of three measures of fit quality; the normalized cross correlation, the difference in intensity of the two signals and the difference in the gradients of the two signals. By doing so, we were able to improve significantly the accuracy with which we could resolve correctly-aligned molecules from an incorrectly-aligned population, even with relatively low labelling efficiencies (Supplementary Figures S6 and S7).

### Locating specific DNA molecules in a sample (pairwise matching)

In a simple implementation of our mapping approach we make a pairwise comparison between an imaged DNA barcode and a barcode generated *in silico* for a genome or genomic feature of interest. Figure 2 gives an overview of the analytical process for matching many DNA barcodes to a single, known reference sequence(s):

1. Each intensity profile is compared, by cross-correlation, with the profile of a known sequence of interest. The relative length of the experimental barcode is compressed (1.5- to 1.7-fold) to allow for optimal matching.
2. The best match between molecules is scored (given an alignment weight) and a histogram for all alignment weights in the dataset is generated.
3. Barcodes with an alignment weighting above a selected threshold value are averaged, and compared to the reference, to confirm the match.

Cross-correlation of the signals of thousands of molecules with this (short) reference sequence in this instance takes around ten seconds (standard laptop computer with 16GB RAM, 3.20 GHz Intel Core i7 processor). Subsequently, an alignment weight is generated and used as a measure of the match quality. Molecules with a weight above a specified threshold are used to generate an average experimental barcode that can be inspected to visually confirm the match to the reference data. This works well in the case where the reference data is a short (say 50–100 kb) sequence of interest, as demonstrated in Figure 2 for the bacteriophage lambda genome. However, the time taken for matching the experimental data scales linearly with the number (total length) of the genomes in the reference library. Hence, for example, running the sample shown in Figure 2 against a library of 2000 virus genomes would take around 5 h. The likelihood of a spurious match occurring also increases with the size of the reference library. Supplementary Figure S8(A) shows the result of matching of individual barcodes from an experiment containing the bacteriophage lambda and T7 bacteriophage genomes against a library of twenty virus genomes. Whilst both the lambda and T7 genomes are well represented in the dataset of correctly matched molecules, neither can be reliably identified by simply assigning barcodes to their best match in the reference database. However, an identification can be made with greater than 80% accuracy by using an appropriate weighting threshold (Supplementary Figure S6, S7 and supporting text) and counting the number of molecules with matches above this threshold for all genomes in the database, Supplementary Figure S8(B).

**Figure 3.**
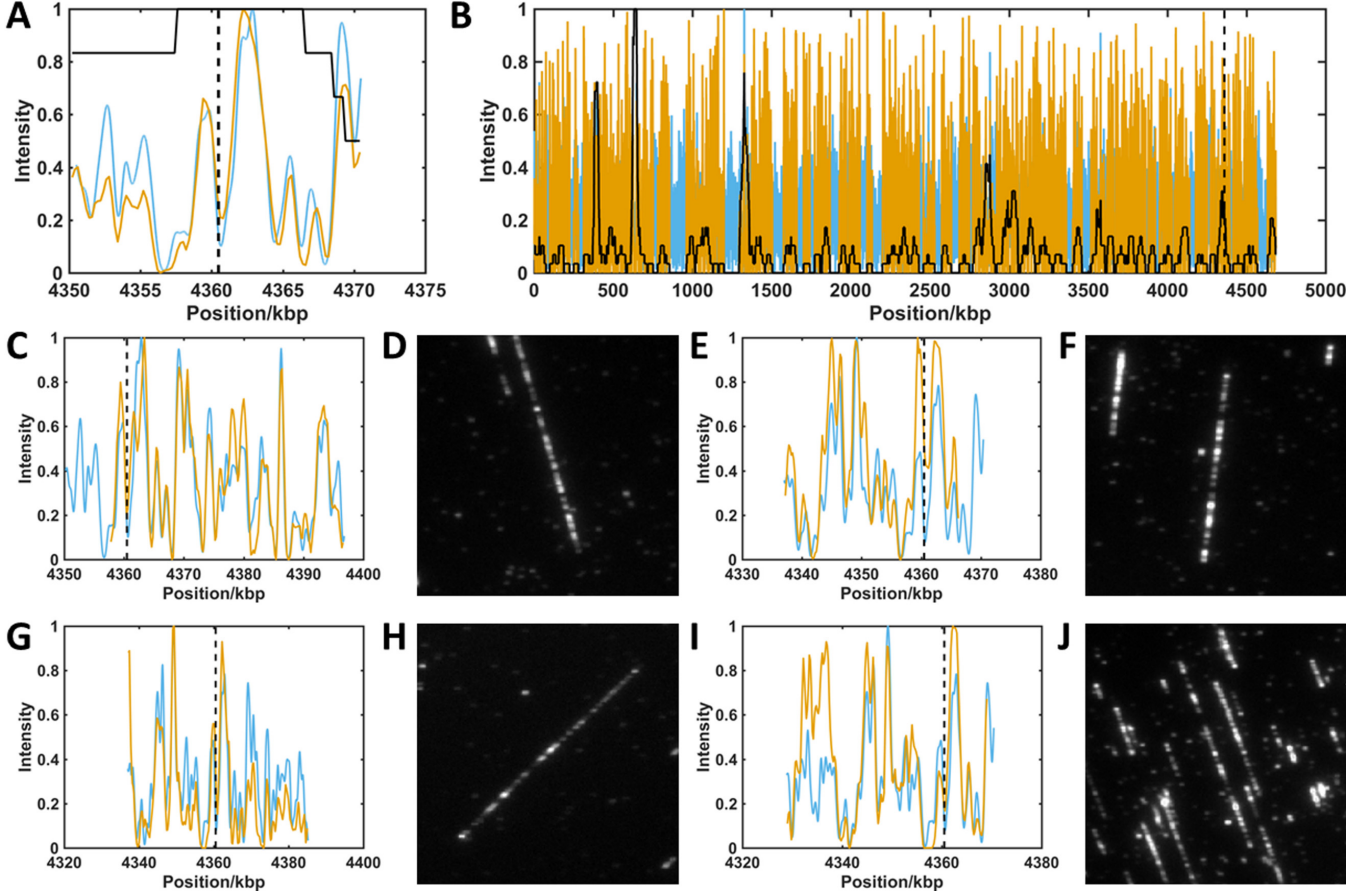
Localization of barcodes containing a CRISPR/Cas9 edit. Red lines show experimental barcode profiles. Blue lines show reference genome profiles. Black dashed lines show the expected position of the edit on the genome. Values in parenthesis below show alignment weight to reference. (**A**) Consensus barcode generated from all barcodes overlapping with at least 25% of the region of interest. A maximum (minimum) of 35 (18) barcodes (solid black line) contribute to the consensus (0.963). (**B**) Consensus of all barcodes aligned to the genome reference. A maximum (minimum) of 168 (0) barcodes (solid black line) contribute to the consensus across the genome (0.815). (**C**, **E**, **G**, **I**) Single molecule barcodes aligned to region of interest (0.866, 0.864, 0.860, 0.831). (**D, F, H**, **J**) Raw images of barcodes (shown in C, E, G and I, respectively) identified as overlapping region of interest.

We now extend this approach to a ‘real-world’ example to identify a specific genomic region of interest. We consider a sample of the *E. coli* strain DH10B (TOP10) genome, which has been edited using CRISPR/Cas9. Although the edit in this case is only 64 base pairs in length (∼30 nm on the stretched DNA) and so cannot be directly visualized using standard optical microscopy, molecules matching the region of interest can be identified from their barcodes and we can subsequently search the original dataset for the images of those molecules, Figure 3. The dataset of filtered barcodes used in this analysis consists of 1629 molecules. The pairwise matching process assigns each to the region of the genome where its alignment weight is the highest. As a result of this, 6 molecules were found to overlay with at least 25% of the 20 kb region of the interest (containing the short genome edit) from the *E. coli genome*. Examples of four of these are shown in Figure 3. Figure 3A shows that the consensus taken from the identified barcodes is in excellent agreement with the reference barcode. Supplementary Figure S9 gives a second example, for the same dataset but with a search for molecules having barcodes consistent with the region of the bacterium's genome carrying the *lacZ* gene. We find fewer barcodes- just two-matching to this region, with a score above the threshold weight, indicating a good match.

Figure 3B shows the alignment of the entire dataset across the *E. coli* DH10B genome. Notably, there are several ‘gaps’ in this barcode alignment, where none of the experimental barcodes are aligned to some regions of the genome. On further investigation, we found that this is due to the reference DNA barcode at these locations, where the matching of barcodes to these regions is less robust, with respect to imperfections in the barcode, relative to other regions of the reference genome. For example, in regions with particularly high densities of M.TaqI sites (bright regions in the barcode) the alignment weighting is relatively unaffected by changes in labelling efficiency, compared to a barcode where the distribution of labels is more uniform across the barcode. Hence, even the best matches in some regions of the genome have alignment weightings that lie below the cut-off threshold that we have applied (uniformly) across the whole genome. Future work will focus on developing a dynamic threshold for any reference genome (a ‘*P*-value’) that allows an unbiased estimation of the appropriate threshold for a ‘good’ match at a given locus. Pairwise matching of the experimental barcodes enables interrogation of a genomic imaging dataset for specific features of interest, based on its underlying DNA barcode (sequence). Currently, the experimental barcodes identified using this approach represent ‘candidate’ barcodes that likely correspond to the identified genomic region but this identification (at the single-molecule level) cannot be regarded as definitive.

### Identification of monocultures of bacteria (pairwise matching)

For a cultured sample, the pairwise matching procedure can be extended to identify viruses and bacteria from a library of known reference genomes. Rather than matching the recorded dataset of DNA barcodes to a single, known reference sequence with a length comparable to that of the barcode, here the dataset is matched to a library containing around one hundred megabases of DNA sequence. Rather than using a specified threshold to locate and visualize the molecules, we rely on the likelihood that, on average, more of the dataset will fit well to the correct genome than will fit spuriously to an alternative, perhaps related genome.

**Figure 4.**
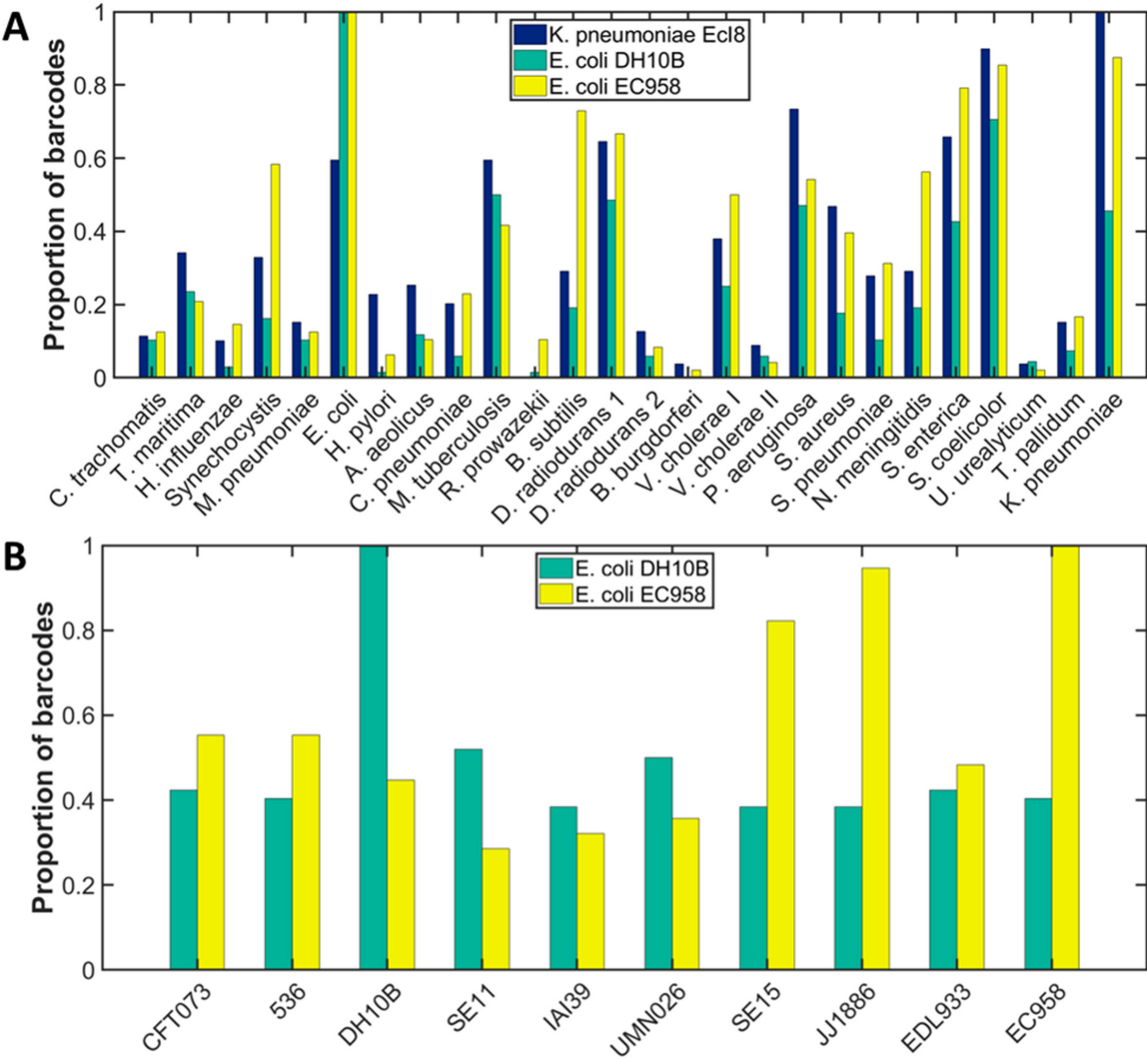
Identification of bacterial DNA by species. Samples of DNA extracted from cultured *K. pneumoniae* strain Ecl8 (blue); *E. coli* strain DH10B (green) or *E. coli* strain EC958 (yellow) were labelled with Atto 647N using methyltransferase-directed labelling (M.TaqI). (**A**) Each barcode from the sample was assigned to the species to which its alignment yielded the highest alignment weight. In each case, a simple count of the number of barcodes in the sample matching a given reference genome in this way, allows the specie of bacterium to be identified. (**B**) Similarly, having identified a species, each barcode is now matched against several bacterial strains and then assigned to the strain to which its alignment yielded the highest alignment weight. Both *E. coli* strains are correctly identified. In both plots the bars are scaled such that the most populated field has a value of 1. Accession numbers for the genomes used and the absolute numbers of barcodes assigned to each are given in the Supporting Supplementary Tables S2 and S3.

We tested this approach on the identification of three cultured isolates of bacteria. The genomes of *E. coli* strain DH10B; *E. coli* strain EC958 and *K. pneumoniae* strain Ecl8 were imaged and the data filtered as described above. We selected a reference ‘database’ of twenty-five bacterial genome sequences, for initial species identification. Each experimental DNA barcode was aligned against this database and is assigned to the genome with which it has the highest alignment weighting, Figure 4A. Since the population of experimental data is imperfect, many incorrect assignments are made (assuming the sample contains only labelled DNA from the cultured organism). However, for all three samples the species with the most barcodes assigned to it is the sample that has been cultured. Furthermore, upon comparison to a database of *E. coli* strains, selected based on their similar phylogeny to the cultured strain, (19) both strains of *E. coli* were correctly identified using this approach. Indeed, the SE15 and JJ1886 strains, which are thought to be closely related to EC958 (belong to the same phylogroup), have a similar number of matched barcodes to the EC958 strain, and all three have significantly more barcodes matched to them than the other, less closely related strains in the plot of ‘matched barcodes’, Figure 4B.

This promising result begins to show how we can use- and what we can reasonably achieve- with the DNA barcode data and current analytical approach. The experimental barcodes are a collection of DNA molecules that are imperfectly labelled, imaged and analysed. As a result, some barcodes, from some regions of the genome can be well-matched to many possible reference genomes. Currently, this is the ‘noise’, inherent in our dataset (Supplementary Tables S2 and S3 describe the number of matched barcodes for the library of genomes used in these experiments). However, cumulatively, using no more than a couple of thousands of experimental barcodes, we are able to generate sufficient numbers of reliable matches to a reference genome that we can give an indicative identity for both species and strain. The alignment weight is not sufficiently discriminating that we can achieve this with a single barcode. Rather, on the balance of probability, from a population of many experimental barcodes, a majority will match to the reference genome in the library that is most closely related to their own.

This comes with some important caveats; the library of ‘other’ genomes we match to is small because running the analysis against a large library would be prohibitively slow (using a personal computer); we apply no threshold to the alignment weight, hence there may be many spurious matches in the dataset (off-target matches may be random); and this process assumes the input DNA is from a purified sample of DNA from a laboratory-grown monoculture. Identification of organisms from their genomes in more complex mixtures requires a more sophisticated analytical routine. We describe such an approach below.

### De novo clustering of similar genomic barcodes (a similarity search)

The mapping dataset offers a unique way to investigate the genomic composition of a sample without prior knowledge of its content. We sought to take advantage of this by developing an approach to cluster, quantify and identify similar molecules in the dataset. In the mapping data, connections between molecules that are highly similar can be made using the alignment weighting. The alignment of every molecule to every other molecule in the dataset allows us to generate an affinity (similarity) matrix for the dataset. The affinity matrix is converted to an adjacency matrix by identifying the most similar matches in the dataset (using the alignment weight) and generating links between them. These communities of closely related barcodes can be used to generate single, consensus barcodes, representative of their community. Such analysis encompasses many thousands of imaged molecules but provides a mechanism for dramatically reducing the size of the dataset and then comparing this reduced representation to a large reference library. For 1000 (∼40 kb) experimental barcodes, the process of generating these communities of similar barcodes takes ∼1 min. In summary, the procedure is as follows:

1. Each DNA barcode compared, by cross-correlation, with the barcodes of every other DNA molecule in the dataset.
2. The resulting matrix of alignment weightings (affinity matrix) is used to link closely related molecules in the dataset (generate communities).
3. An average barcode is generated for each community and this is compared to a large library of reference genomes to identify the community.

**Figure 5.**
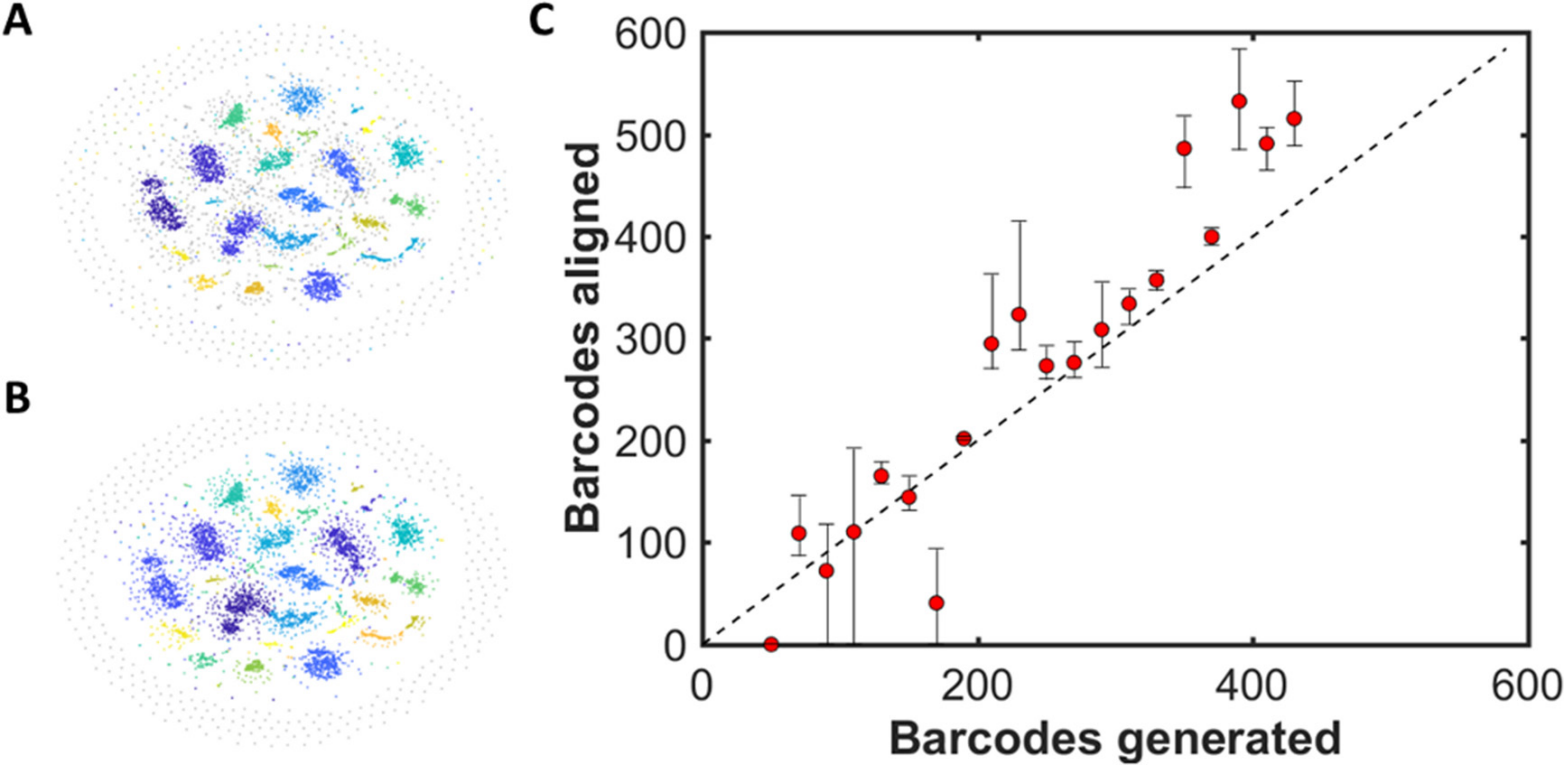
Community detection for barcodes generated in silico. For each of 20 bacteriophage genomes, between 50 and 430 barcodes were generated, with 50% labelling efficiency (coloured dots). 1200 randomly generated barcodes were also added to the simulation (grey dots). (**A**) t-SNE visualization of the network of communities generated from the synthetic data. Each colour represents a different genome with the grey dots representing randomly-labelled DNA barcodes. (**B**) t-SNE visualization of the communities detected in the dataset. Each colour represents a community of similar DNA barcodes. Note that communities tend to absorb similar random barcodes. As expected, a ring of predominantly random barcodes, which are poorly matched to all other barcodes in the sample, appears on the periphery of the t-SNE visualization. (**C**) Plot showing the mean number of barcodes matched against those generated. Data was shuffled and aligned five times with error bars showing the maximum and minimum number of barcodes assigned to a given genome for the five repeats.

We validated this approach using a total of 6000 DNA barcodes generated in silico; 4800 of these are from a selection of 20 viral genomes and the remaining 1200 are randomly generated to represent, for example, contamination of the sample or poorly labelled DNA molecules. The analysis groups these molecules into a series of distinct clusters. Similarity within the dataset is visualized using a tool for dimensional reduction, t-SNE (20), in Figure 5. Note that, although we use t-SNE to visualize the data, the communities of similar barcodes are generated based on the affinity matrix (which is difficult to visualize), not the t-SNE plot. The t-SNE plot displays each DNA barcode as a single dot and clusters similar barcodes closely. Around the periphery of the plot is a ring of barcodes that are equally dissimilar to all other barcodes in the dataset (the predominantly grey colouring of the dots indicates that these are almost exclusively the randomly-generated DNA barcodes in the dataset). The consensus barcodes for each detected community were generated and subsequently aligned against a library of 1994 phage genomes, a process that took around 1 h to complete. Figure 5C and Supplementary Table S2 summarize the output of these alignments for five shuffles of the dataset. Randomly re-ordering (shuffling) the dataset changes the relative positions of any two molecules in the affinity matrix for that dataset. We use this to ensure that we recover similar communities for each shuffle and, hence, can be confident that the process we use for constructing affinity and adjacency matrices is robust, with respect to the order of the dataset.

Figure 5 shows that, using data generated *in silico*, 18 of the 20 bacteriophage genomes that were introduced to the mixture can be consistently identified across all five repeats of the analysis. Of the other 1974 genomes in the library, no incorrect (false positive) matches were made. The KBNP1711 genome is not identified in any of the analyses and we attribute this to the inherently low score our alignment weighting gives to good matches with this genome. Other factors, such as absolute and relative barcode numbers, as well as coverage, also play a role in determining the efficacy of the analysis detecting a given genome. Also, note that the impact of the randomly generated barcodes on the analysis limits an absolute, quantitative analysis of the data at this point. Such barcodes represent, for example, a contaminating population of DNA in the dataset or poorly labelled DNA molecules. The detected communities (Figure 5B) invariably expand to absorb a handful of these molecules, such that we generally overestimate the number of barcodes belonging to a given community in the analysis.

### Identifying viruses against large quantities of host cell genome background

To test this approach experimentally, we sought to apply it to identify viral infections of cells. In a first example, we doped a known virus genome (bacteriophage lambda) into a sample of bacterial genomic DNA (*E. coli* ER2566), which was labelled and imaged, as described earlier. We mixed the sample at a ratio of 1:4 (weight for weight virus: bacterial genomic DNA) to mimic the expected copy number- around 20 copies per cell- of such an infection.

**Figure 6.**
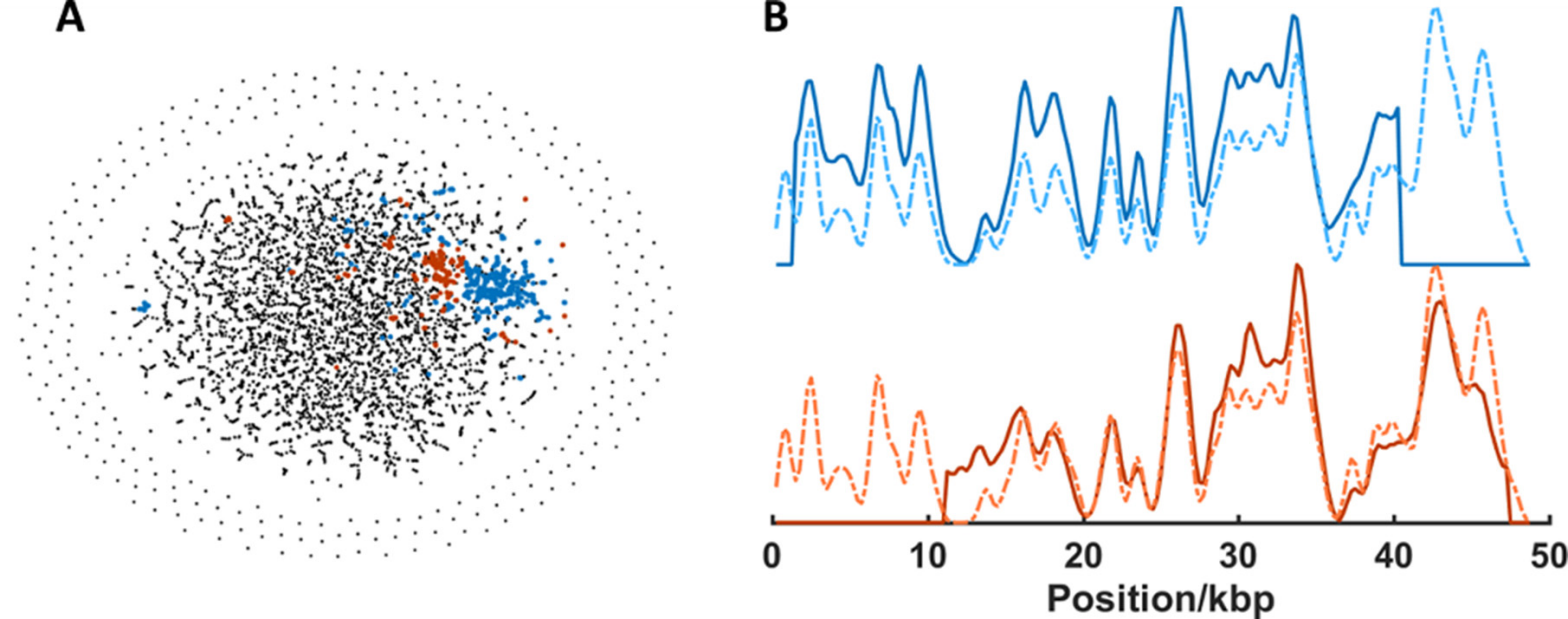
Identification of Lambda bacteriophage DNA in genomic mixture by *de novo* separation and alignment of experimental barcodes. (**A**) t-SNE visualization of the communities generated from the adjacency matrix. Community detection gives rise to two clusters (blue and red points) which are matched to the lambda phage genome. A total of 475 of 5114 barcodes are in these two clusters. (**B**) Consensus (average) barcodes (solid lines) generated from the blue and red clusters and their alignment to the bacteriophage lambda reference genome (dotted lines).

Figure 6 shows a clear and unambiguous identification of the phage lambda genome from the mixed sample. In this case, we extracted 5114 barcodes from the imaging dataset, of which 475 were clustered and identified as the lambda phage genome. Figure 6A shows the tSNE visualization derived from the affinity matrix and the two clusters of similar barcodes that are identified as the bacteriophage lambda DNA highlighted in red and blue. The process of identification, using a library of 2000 phage genomes took 47 min to run on a standard laptop computer (16GB RAM, 3.20 GHz Intel Core i7 processor). The t-SNE visualization of the data in Figure 6 is typical of an experimental dataset, where (amongst other factors) labelling efficiency, impurities in the sample and aggregation of DNA barcodes give rise to larger, more diffuse communities than we observed for the simulated dataset. Figure 6B shows that this noise in the dataset has no significant impact on our ability to accurately identify a simple virus genome from a library of possibilities, using the consensus barcode of a given community. In total, 2 out of 41 clusters were identified as the bacteriophage lambda genome. Quantitatively, the absolute number of lambda phage genomes in the sample is underestimated, with 1 in 10 of the barcodes attributed to the bacteriophage lambda, whereas we introduced approximately one in five to the mixture.

**Figure 7.**
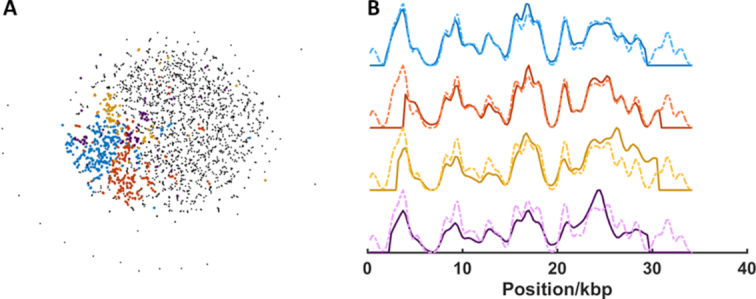
Identification of human adenovirus A DNA sample by separation, de novo alignment and assignment of consensus barcode to reference library. (**A**) t-SNE visualization of the communities generated from the adjacency matrix. Community detection gives rise to four clusters (blue, red, yellow and purple points) which are matched to the human adenovirus A genome. 669 of 1986 barcodes (4 from 21 clusters) are assigned to adenovirus A DNA. (**B**) Consensus (average) barcodes (solid lines) generated from the blue, red, yellow and purple clusters and their alignment to the human adenovirus A reference genome (dotted lines).

We extended this model study to investigate an infection of a population of cultured human (HeLa) cells, infected with human adenovirus A (type 12), 72 h, post-infection. The mixed sample of human and viral DNA was fluorescently labelled, deposited and imaged. The barcodes extracted from these images (1986) were compared to one another, creating an affinity matrix from which 21 communities were identified. Consensus barcodes from these were compared to a database of 128 reference barcodes of vertebrate viruses. A single virus (human adenovirus A) was identified from the sample, in ∼10 min. Figure 7 shows at tSNE visualization of the four communities of barcodes from the sample that are identified as the human adenovirus A and their associated consensus barcodes. Note that each community describes a slightly different consensus barcode, Figure 7B. Supplementary Figure S10 shows exemplars of individual virus DNA barcodes identified in this sample.

In the two above examples, each cell in the sample contains several tens of copies of an invading genome. We sought to explore the limits of our approach by applying this to the identification of (relatively low copy number) plasmids in bacterial cultures. Initial simulations of mixed samples of genomic DNA and plasmid DNA showed promising results, even where we introduce appropriate levels of experimental imperfections to the synthetic dataset. However, initial experiments using this approach have proven unsuccessful in reliably identifying large plasmids in bacterial cultures. This we attribute to their low copy number in bacterial cells. Indeed, if we reduce the number of copies of a plasmid to less than five per cell, simulations show that it is (unsurprisingly) difficult to identify a community of barcodes that can be mapped to a specific plasmid. However, since the sequence of interest is known in this case, a simple pairwise alignment of the dataset to a reference (plasmid) barcode can be used and has yielded a handful of candidate barcodes with high alignment weighting to the resistance plasmid (Supplementary Figures S11–S13). Further investigation is required to confirm the identity of these molecules, yet this preliminary result suggests that the identification of a small number of molecules of interest from a large sample is possible. Such an approach could be twinned with super-resolution imaging in the future to better resolve the barcodes of such a subset of candidate molecules.

## CONCLUSION

We have provided a basis for the use of DNA barcodes as a tool for visualization of the sequence of whole genomes. We have collected multiple datasets containing gigabases of DNA, each of which is of the order of 120MB in size. We have shown that this data can be searched for a genomic region of interest, used to search a library of genomes for matching organisms and that similar molecules within the dataset can be identified and clustered to identify viral infections against the background of host genomic material. In the future, work will focus on exploiting the strength of these optical measurements to allow multiplexed studies of both sequence and ‘event’. Indeed, we believe that this unique view of the genome will enable a new perspective, i.e. one with sequence context, on events and processes, such as replication (in combination with labelling of replicated DNA using halogenated (21) or alkyne-functionalized (22) nucleotide analogues), (fluorescently-tagged) drug or protein binding or genomic editing across whole genomes, at the single-molecule level.

## DATA AVAILABILITY

The datasets used for analysis in this study and an annotated version of the Matlab code we used to extract, process and analyse DNA barcodes are available at edata.bham.ac.uk, DOI: 10.25500/eData.bham.00000255.

## Supplementary Material

gkz212_Supplemental_File
