# Supplementary material for: DNA barcodes for rapid, whole genome, single-molecule analyses
Source: Nucleic Acids Res. 2019 Mar 28;47(12):e68. doi: 10.1093/nar/gkz212 (PMC6614835; doi:10.1093/nar/gkz212)
Supplement: gkz212_Supplemental_File [file gkz212_supplemental_file.pdf]

# DNA barcodes for rapid whole genome, single-molecule analyses

Nathaniel O. Wand[1], Darren A. Smith[1], Andrew A. Wilkinson, Ashleigh E. Rushton, Stephen J. W. Busby[2], Iain B. Styles[3] and Robert K. Neely[1,*]

[1] School of Chemistry, University of Birmingham, Edgbaston, Birmingham, B15 2TT, UK
[2] School of Biosciences, University of Birmingham, Edgbaston, Birmingham, B15 2TT, UK
[3] School of Computer Science, University of Birmingham, Edgbaston, Birmingham, B15 2TT, UK

### Methyltransferase-directed DNA labelling

DNA labelling was carried out as described in the main manuscript. Below are the structures of the compounds used. A commercial kit for performing a similar reaction M.TaqI is available from Chrometra (MTaze-azide).

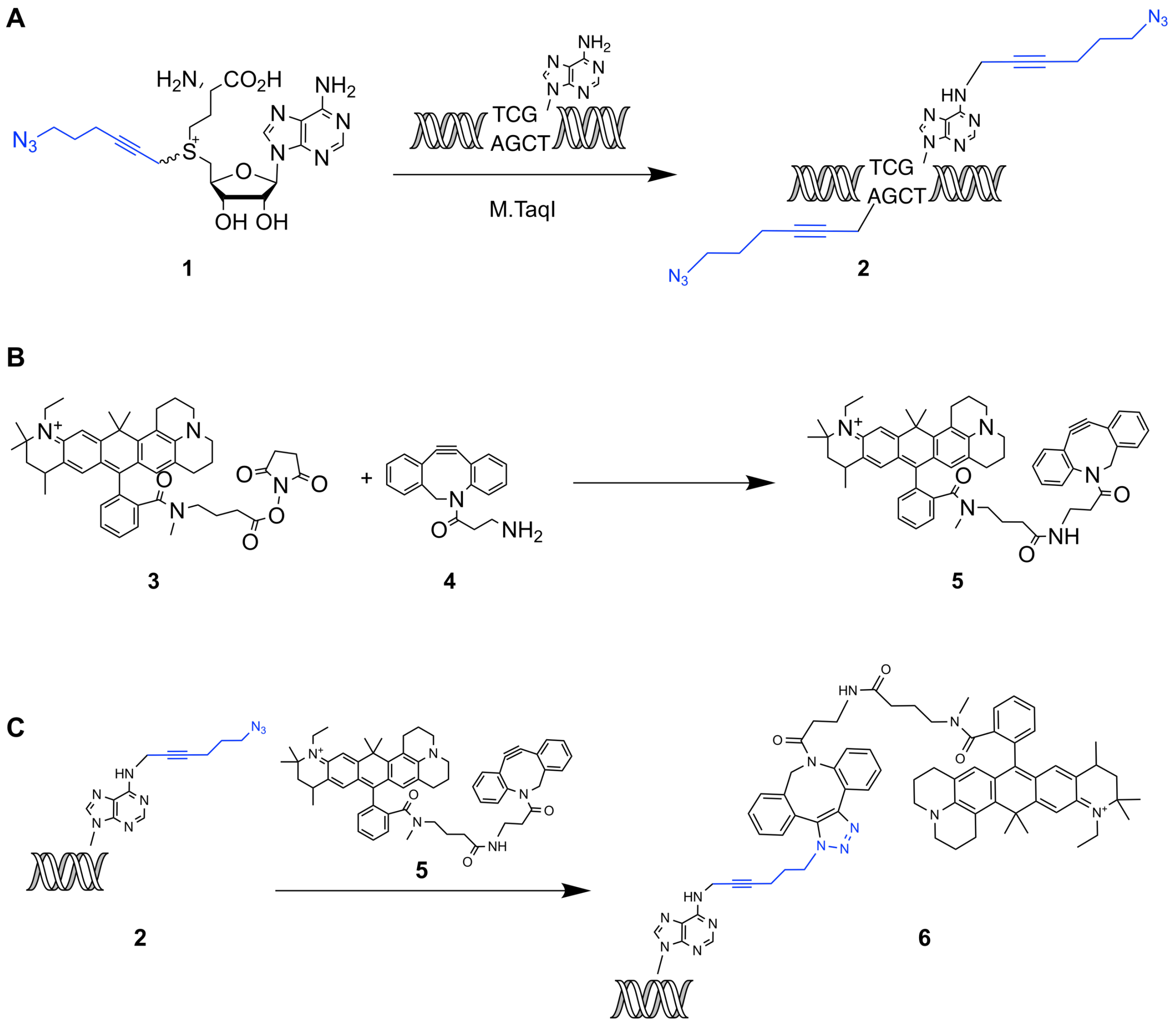


*Figure S1:* A) Transalkylation of DNA at the adenine of sites reading 5'-TCG**A**-3' (2) using the AdoHcy-Azide cofactor (1), catalyzed by the M.TaqI enzyme. B) Formation of dibenzylcyclooctyne (DBCO)-Atto647N (5) from Atto647N-NHS ester (3 and DBCO-amine (4). C) Coupling of Atto674N-DBCO (5) to azide-labelled DNA (2) to give Atto647N-labelled DNA at M.TaqI sites (TCGA) (6).

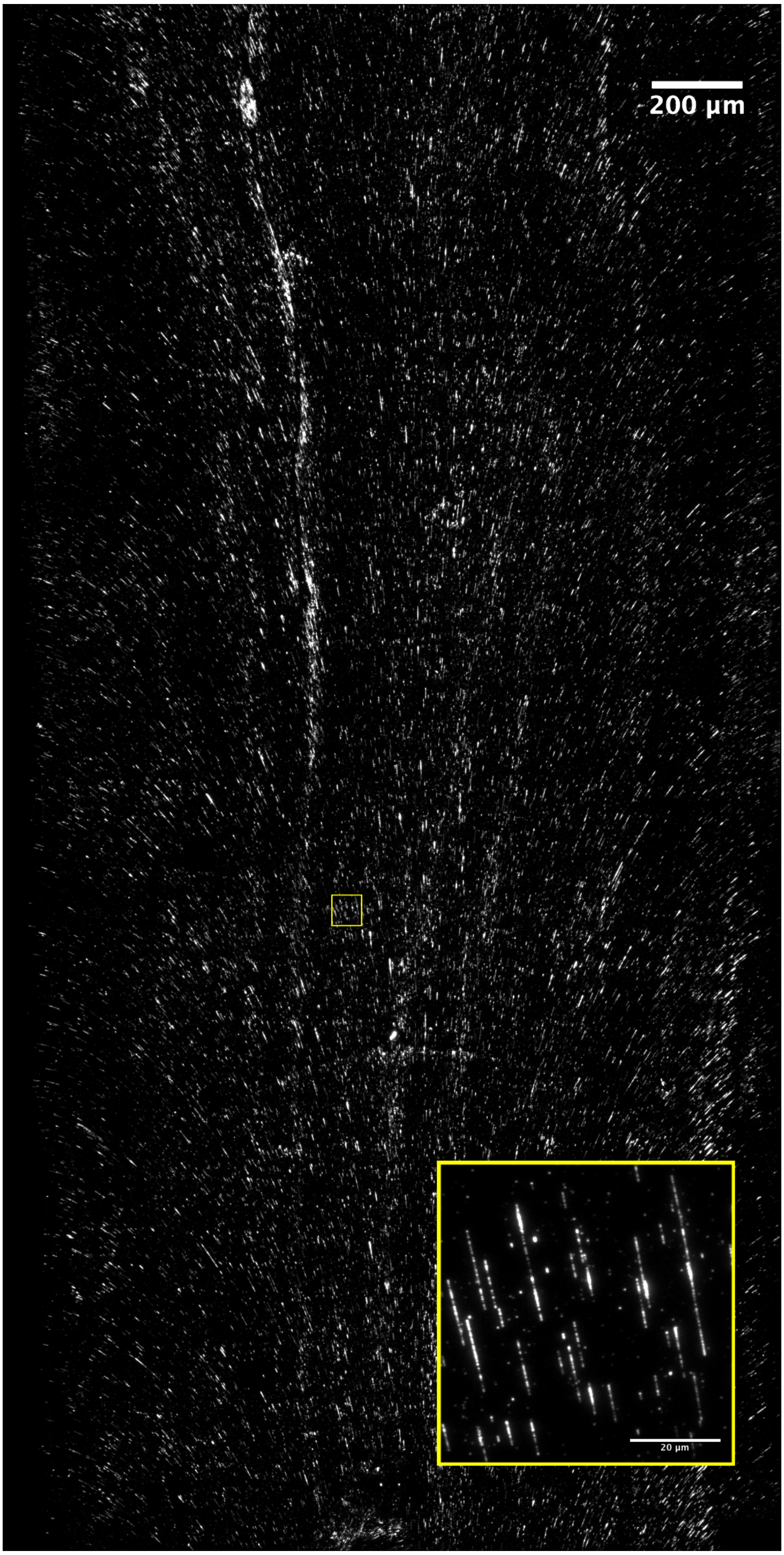


*Figure S2:* An overview of a subset (20x40 tiled images) of the dataset collected for a sample of the *E. coli* genome. Images have had their background subtracted to improve the tiling.

**Automated extraction of DNA barcodes from images**

We developed and applied (Matlab 2016b) the following procedure for the automated identification and extraction of DNA from combing experiments:

1. Estimate direction of combing (theta) using the Hough transform.
2. Smooth lines in the identified direction using convolution with a Gabor filter
3. Detect edges of DNA molecules with Sobel edge detection.
4. Use the edges to define coordinates for intensity profiles
    - Image dilation in direction of theta to make a continuous line
    - Group edges by connectivity (i.e. define each edge)
    - Extract ends of edges
    - Merge close ends to define lines along DNA molecules
    - Extract intensity along line
    - Extract and store DNA length. Convert to estimate of DNA length in base pairs (1.93 bp/nm).

The procedure is summarised in Figure S3.

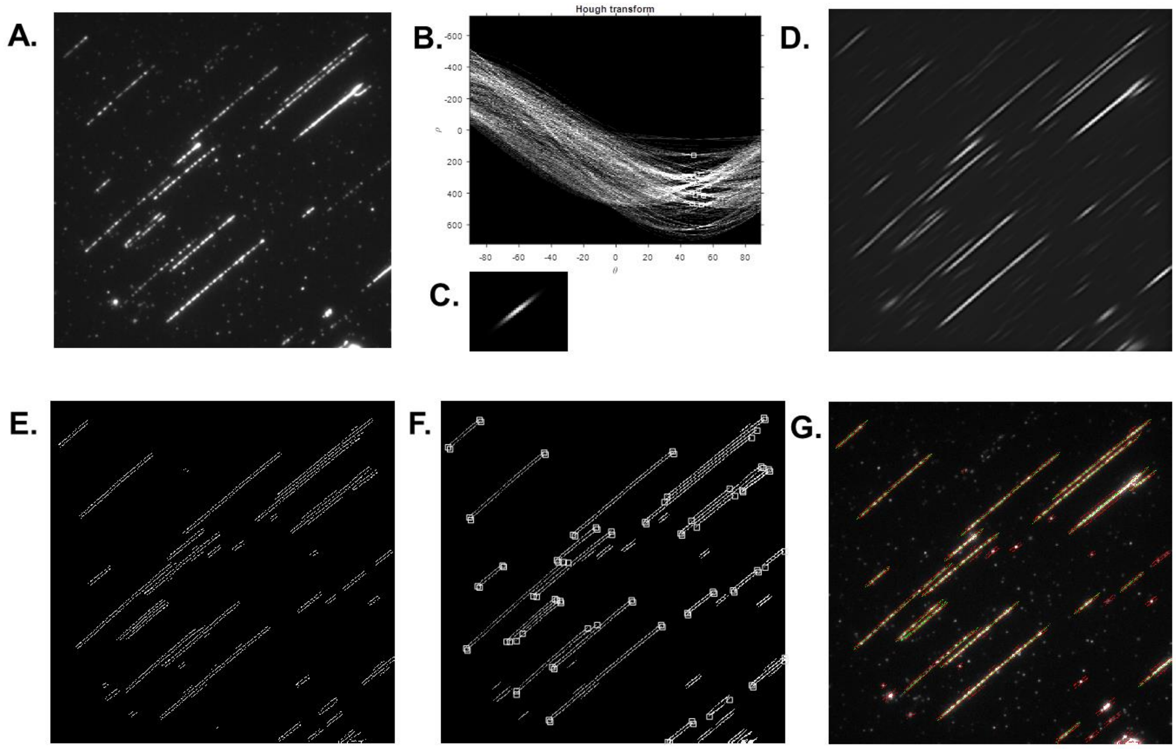


*Figure S3:* Automated extraction of intensity profiles. A) Typical combing image. B) Hough transform used to estimate direction of combing. 10 peaks are selected and the median used to define theta. C) A Gabor filter in the direction of theta is created and a convolution with A) gives D). E) Edge detection on D) using the Sobel method and an automatically detected threshold. F) Edges are dilated and connectivity used to define the ends of lines. When two pairs of end points are close a line is drawn between them. G) This line is shown in green, edges in red.

**Alignment of DNA barcodes to reference data**
An overview of the alignment procedure is given below:

1. Generate reference barcode
   - Reference sequence is imported from FASTA and converted to numeric form (A=1, C=2, G=3, T=4)
   - Convert to labelled sequence. Labelled bases=2, unlabelled bases=0. E.g. for M.TaqI the A of TCGA=2, all other bases=0.
   - Convolution with PSF, select sigma from 250-400bp
   - Sample every N base pairs
2. Generate experimental barcode
   - For each extracted, scaled DNA barcode:
   - Sample every N base pairs
   - Store forward and reverse barcodes
3. Cross-correlation with reference barcode to define best stretch
   - Use normalised cross correlation and test for 90%-110% of estimated stretch
   - Maximise normalised cross correlation to define best stretch and orientation
4. Align fragment
   - Maximum normalised cross correlation gives corresponding displacement
   - Use displacement to align stretched and oriented fragment along reference genome
5. Determine alignment weight
   - Determine mean difference in intensities and the mean difference in the gradients of the aligned barcodes and reference genome
6. Repeat for each DNA fragment

Full details of the procedure for aligning DNA barcodes are given below.

**Weighted cross-correlation**
A candidate barcode profile (*Y*, length *k* pixels) is first normalised using mean ($\mu$) and standard deviation ($\sigma$) values (**Error! Reference source not found.**A and C).

$$Y_N = \frac{Y - \mu_Y}{\sigma_Y}$$

The (sampled) reference profile (*I*) is normalised by moving mean and moving standard deviation values, where the span of the moving window is equal to the size of the barcode being aligned (*k*).

$$I_N = \frac{I - \mathrm{mov}(\mu_{\mathrm{I}}, k)}{\mathrm{mov}(\sigma_I, k)}$$

Moving values are used to reduce the effect of differences between local and global intensity values. High intensity regions of the reference profile would otherwise obtain consistently higher cross-correlation values and thus barcodes would be more likely to align to these regions. A slight distortion is introduced into the profile of the reference barcodes after normalisation using moving values and so a perfect match (which would be expected from a fragment cut directly from the reference) is no longer possible. It should be noted, however, that this distortion is negligible compared to the

corruption caused by noise, imperfect and offsite labelling of experimental barcodes. Cross-correlation values (*CC*), calculated with MATLAB's `xcorr` function, are normalised by the size of the barcode such that an autocorrelated barcode should obtain a value of 1.

$$CC = xcorr(Y_N, I_N)/k;$$

MATLAB's `xcorr` function calculates cross-correlation via

$$CC(m) = \hat{R}_{xy}(m - N), \qquad m = 1,2,\ldots,2N-1$$
$$\hat{R}_{xy}(m) = \begin{cases} \sum_{n=0}^{N-m-1} x_{n+m} y_n^* & m \geq 0 \\ \hat{R}_{xy}^*(-m) & m < 0 \end{cases}$$

A mask (M) is created to discourage alignment beyond the edges of the reference profile (barcodes should not contain sequence information that is not within the reference). Practically the mask is created by cross-correlation of binary versions on the barcode and reference profiles, normalised by the length of the barcode (*k*), such that the mask has a value of 1 where there is full overlap between barcode and reference and attenuates linearly to a value of zero as the barcode extends beyond the reference.

$$Y_B(i) = \begin{cases} 1, & Y(i) > 0 \\ 0, & Y(i) = 0 \end{cases}$$
$$I_B(i) = \begin{cases} 1, & I(i) > 0 \\ 0, & I(i) = 0 \end{cases}$$
$$M = xcorr(Y_B, I_B)/k;$$

Weighted cross-correlation values are calculated by multiplying the original cross-correlation vector (*CC*) with the mask. The barcode is aligned to the reference genome at the position which has the highest weighted *CC* value.

$$CC_W = CC \cdot M$$

**Modulating alignment weight**
Despite the use of moving mean standard deviation values, the barcodes still have a higher propensity to align to relatively bright (higher intensity) regions of the genome. This is particularly a problem when the genome is large (since there is more likely to be relatively bright and dim local regions). To try and reduce effects of the artificial benefit gained by aligning to a brighter region, the weighted cross-correlation value is further modulated by two further measures: the mean difference between barcode and reference profile intensities at the aligned position and the mean difference in the gradients of barcode and reference profile intensities at the aligned position. These two measures are calculated in the following manner.

First the intensity profile of the reference at the aligned position ($I_k$, length k) is normalised by the local maximum.

$$I_a = \frac{I_k}{\max(I_k)}$$

The absolute intensity difference between the barcode and the reference profile is then calculated.

$$\Delta I = |I_a - Y|$$

The intensity difference contribution to the final weight (*U*) is then calculate by subtracting twice the mean of these values from unity

$$U = 1 - 2 \cdot \mu_{\Delta I}$$

Thus, a barcode with well-matched intensities to the reference will obtain a *U* value close to 1 while a poorly matched barcode will have a *U* value close to zero. In the extreme case of an anti-correlated barcode (i.e. $Y = -I_a, \Delta I = 2I_a$) the value of *U* will be −1 (since $I_a$ values range between 0 and 1 and are approximately randomly distributed, thus having a mean of ~0.5).
Local gradients are estimated by calculating the difference between neighbouring profile points.

$$GradY(i) = Y(i) - Y(i+1), \qquad GradI(i) = I_a(i) - I_a(i+1)$$

These gradient values are normalised by twice the maximum absolute value obtained.

$$GradY_N = \frac{GradY}{2 \cdot \max(GradY)}, \qquad GradI_N = \frac{GradI}{2 \cdot \max(GradI)}$$

The absolute difference between the barcode and the reference profile gradients is then calculated.

$$\Delta Grad = |GradI_N - GradY_N|$$

The gradient difference contribution to the final weight (*V*) is then calculate by subtracting twice the mean of these values from unity

$$V = 1 - 2 \cdot \mu_{\Delta Grad}$$

Similar to *U*, the value of *V* can range (approximately) between 1 and −1. The final weight (*W*) for the alignment is then calculated as the mean of $CC_W$, *V*, and *U*

$$W = \frac{CC_W + U + V}{3}$$

Note that the modulating values (*U* and *V*) have no effect on the alignment position of the barcode; they are only used to improve the reliability of the alignment quality (as compared to how someone might visually judge the alignment quality, which is based on both relative scale (intensity difference, *U*) and shape (gradient difference, *V*)).

**De novo identification of similar DNA barcodes**
Identification of populations of similar DNA barcodes within a sample was achieved by generating an affinity matrix for the experimental dataset. For every DNA molecule in the sample an alignment weight to every other DNA molecule in the sample was generated. To order the affinity matrix, i.e. to convert it to an adjacency matrix, the alignment weight of the molecules was used to define connections (edges) between similar molecules (nodes). The adjacency matrix is refined by removing edges between nodes that share few connections and, conversely, adding edges between nodes with many connections. This sorted adjacency matrix is used to describe the connectivity between DNA molecules in the sample data. Communities of similar molecules are identified using Matlab's GCModulMax2 function, part of the 'Community Detection' toolbox. For each identified community, a consensus (average) DNA barcode is generated for further analysis.
A visualization tool, t-Distributed Stochastic Nearest Neighbour Embedding (t-SNE) was used to visualise clusters of similar DNA molecules in the sample.

## *In silico* characterization of the accuracy of map alignment

### Monte Carlo simulations

Monte-Carlo simulations were used to assess the sensitivity of matching mapping data to a range of experimental variables. The simulations explore the experimental variation by generating imperfect barcodes that are described by a range experimental parameters, with some reasonable boundaries, as described in Table S1. 100 barcodes are generated for each of 5000 sets of parameters and aligned to the reference barcode. The position from which the barcode was generated is compared to the aligned position and where overlap between the generated and fitted position is greater than 98% the barcode is considered to be correctly aligned. This means for each set of parameters the number of correctly aligned barcodes can be determined.

*Table S1: Experimental parameters used for in silico generation of barcodes for Monte-Carlo simulations.*

| Variable | max | min |
|---|---|---|
| Genome sequence | n/a | n/a |
| No of fragments to be generated | 100 | 100 |
| Labelling efficiency | 1 | 0.1 |
| Probability of non-specific labelling (per base pair) | 0.01 | 0 |
| Variation in fluorophore intensity | 0 | 1 |
| Minimum length of fragment (in base pairs) | 30000 | 10000 |
| Maximum length of fragment (in base pairs) | min+10000 | min+10000 |
| Variation in stretching | 0.2 | 0 |
| Average pixel size (base pairs per pixel) | 500 | 100 |
| Variation in pixel size (dependent on DNA orientation) | 0.2 | 0 |
| Variation in pixel sampling (in base pairs) | 0.2 | 0 |
| Magnitude of noise | 1 | 0 |
| Standard deviation for reference PSF (in base pairs) | n/a | n/a |
| Standard deviation for experimental PSF (in base pairs) | 500 | 250 |
| Variation in experimental PSF | 0.5 | 0 |

A 2D histogram can be produced for each parameter, plotting the number of correctly fitted fragments against the value of the parameter.

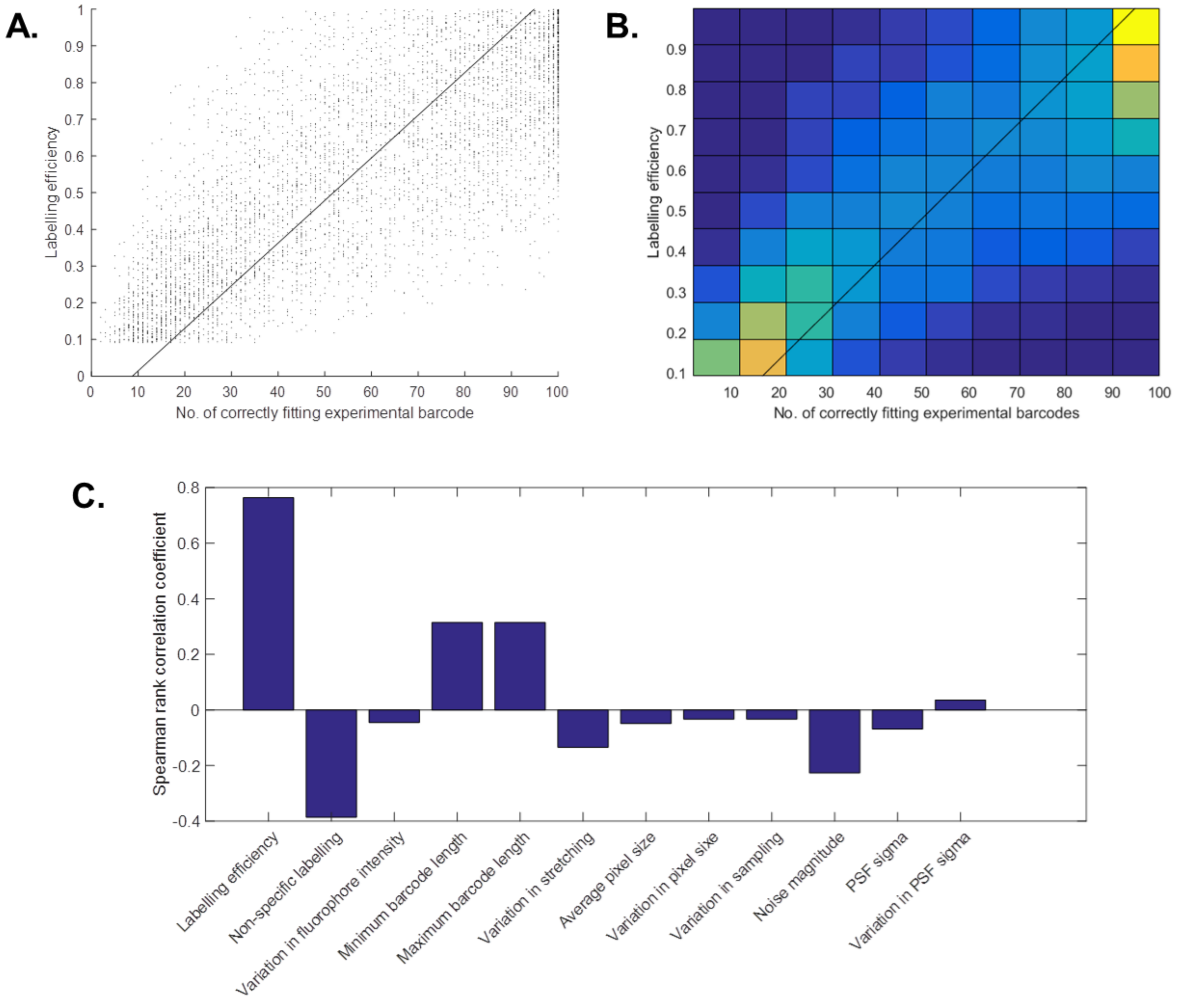


*Figure S4: Monte-Carlo simulation to test sensitivity of experimental variables. 5000 sets of variables were run for 100 fragments each. Experimental barcodes were generated and aligned from/to the bacteriophage T7 genome. A) Example scatter plot for the variation in the labelling efficiency. Each point in the scatter plot in represents a single Monte-Carlo run for a specific set of parameters. B) Dependence of number of correctly fitted barcodes on labelling efficiency, visualised as a 2D histogram. For any given variable, the line of best fit in this plot shows the sensitivity of the number of correctly fitted barcodes to that experimental variable. C) Comparison of the dependency of number of correctly fitted barcodes on the tested experimental variables, quantified using the Spearman rank correlation coefficient for each.*

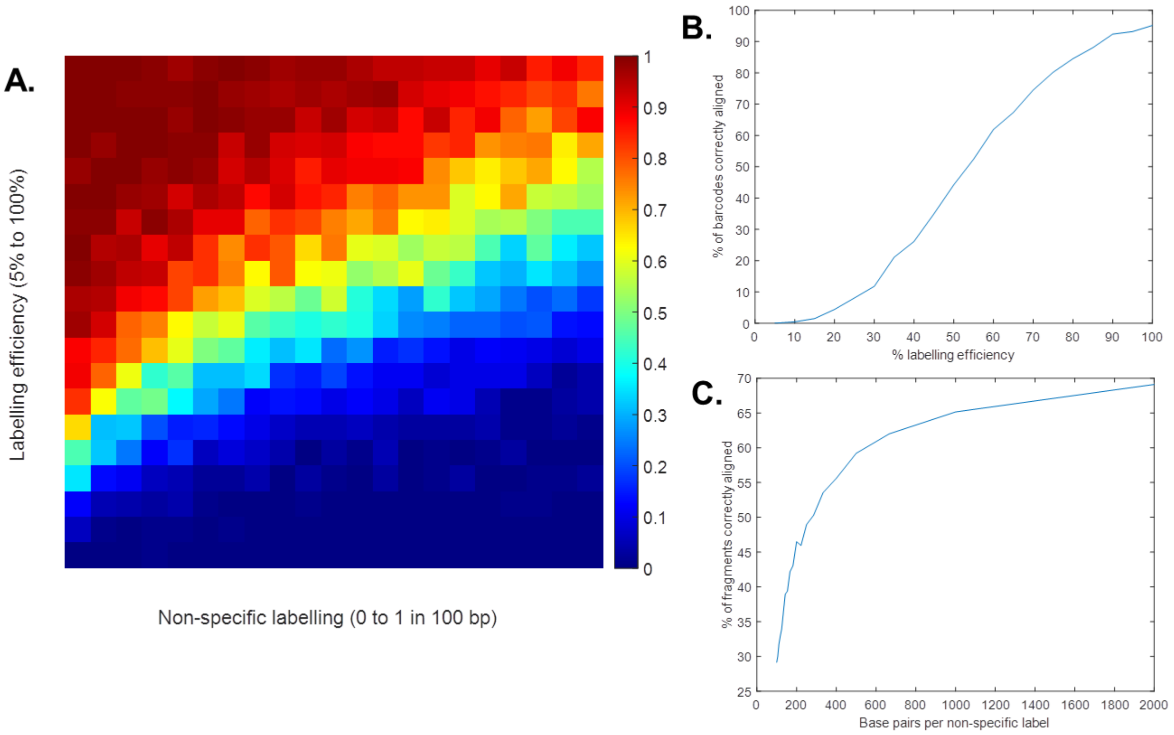


*Figure S5: Simulating effect of labelling efficiency and non-specific labelling on alignment of DNA barcodes to/from E. coli K-12. M.TaqI-directed labelling is simulated, with parameters given in Table S1. A) Simulation of the dependence of the fraction of barcodes (blue-red colour scale) reliably aligned to a reference genome on the efficiency of labelling and the non-specific labelling frequency ( 0 to 1 labels per 100 base pairs). 10 barcodes are generated and aligned per pixel and colour indicates the fraction of these that were correctly aligned. B) Average number of barcodes correctly aligned against labelling efficiency. C) Average number of barcodes correctly fitted against the frequency of offsite labels.*

**Distinguishing correct from incorrectly aligned molecules**

We apply a threshold that defines the weighting above which molecules are described as ‘correctly aligned’ using our fitting procedure. In order to rationalize the choice of threshold value, we used the *in silico* dataset we generated and optimized the accuracy for the threshold (i.e. its ability to discriminate between correctly and incorrectly aligned barcodes) as follows:

$$accuracy = \frac{number\ of\ true\ positives + true\ negatives}{total\ number\ of\ barcodes}$$

where true positive/ negative describe barcodes correctly identified as being in the correct/ incorrect locations. For any given test dataset (generated *in silico*), we are able to generate a plot of accuracy against the threshold used in our map alignment procedure.

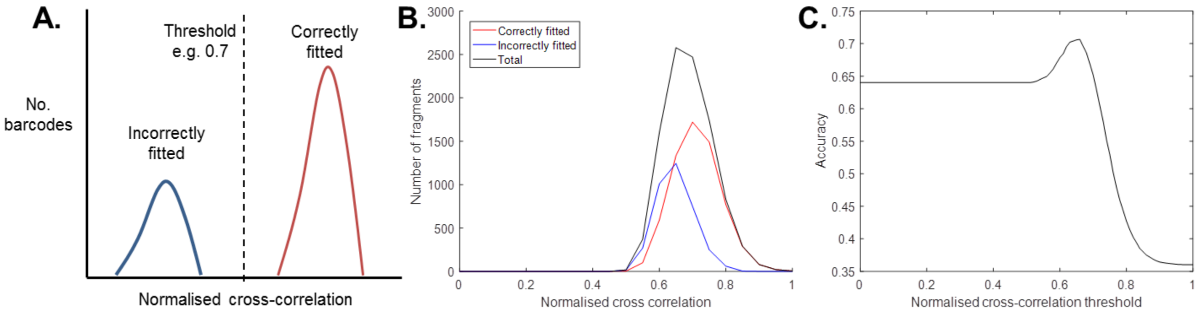


*Figure S6: Normalised cross-correlation as a measure of alignment accuracy. A) Ideal separation by normalised cross correlation. Correctly fitted barcodes (red) will have a higher normalised cross correlation than those that are incorrectly aligned (blue). A threshold can be used to discriminate with 100% accuracy. B) Example for barcodes generated from and aligned to E. coli K-12, with 40% labelling efficiency. There is a large amount of overlap between correctly (red) and incorrectly (blue) fitted barcodes, meaning that for an accurate alignment, a high threshold must be set, thereby discarding much of the dataset from subsequent analysis. C) The accuracy of separation at different normalised cross-correlation thresholds, for data in B).*

Accuracy is improved by combining the measures included in our alignment weighting (average intensity and average slope) with the cross-correlation, Figure S6. Using this alignment for *in silico* data against the *E.coli* genome, we can estimate a reasonable threshold for the fitting procedure of between 0.7 and 0.8.

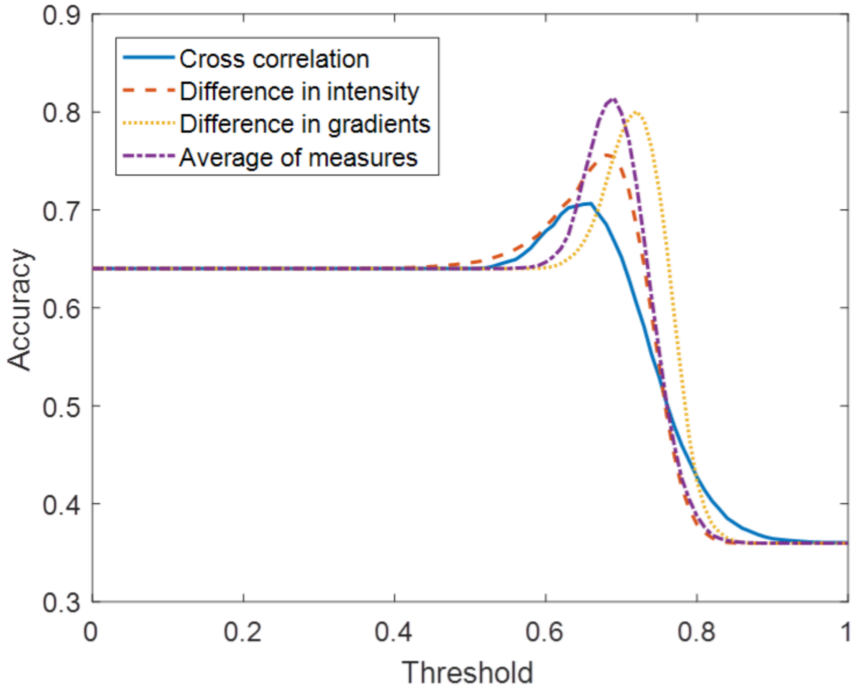


*Figure S7: The accuracy of separation using alternative measures. 10,000 barcodes generated from and aligned to E. coli K-12, with 40% labelling efficiency. Correctly and incorrectly aligned barcodes are separated by several measures, using thresholds ranging from 0 (no alignment) to 1 (perfect alignment). Measures include normalised cross-correlation (blue); difference in intensity (red); difference in gradients (yellow); and an average of all three measures (purple).*

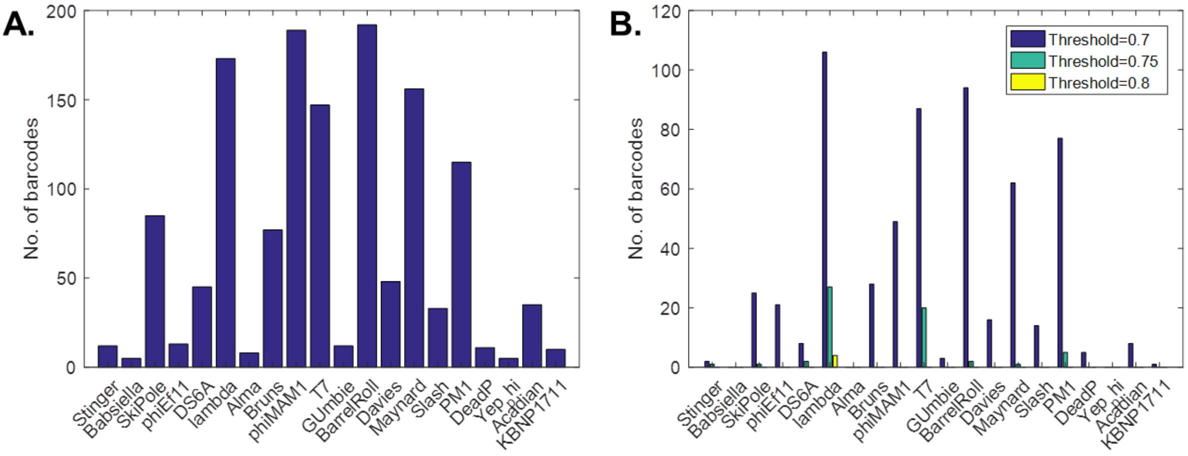


*Figure S8: Identification of bacteriophage DNA. 1756 experimental barcodes from a mixed T7/lambda sample. A) Each experimental barcode was assigned to the phage to which its alignment yielded the highest alignment weight. Note that lambda and T7 cannot be readily identified. B) The number of experimental barcodes aligned to each reference genome with an alignment weight greater than 0.7 (blue), 0.75 (cyan) or 0.8 (yellow).*

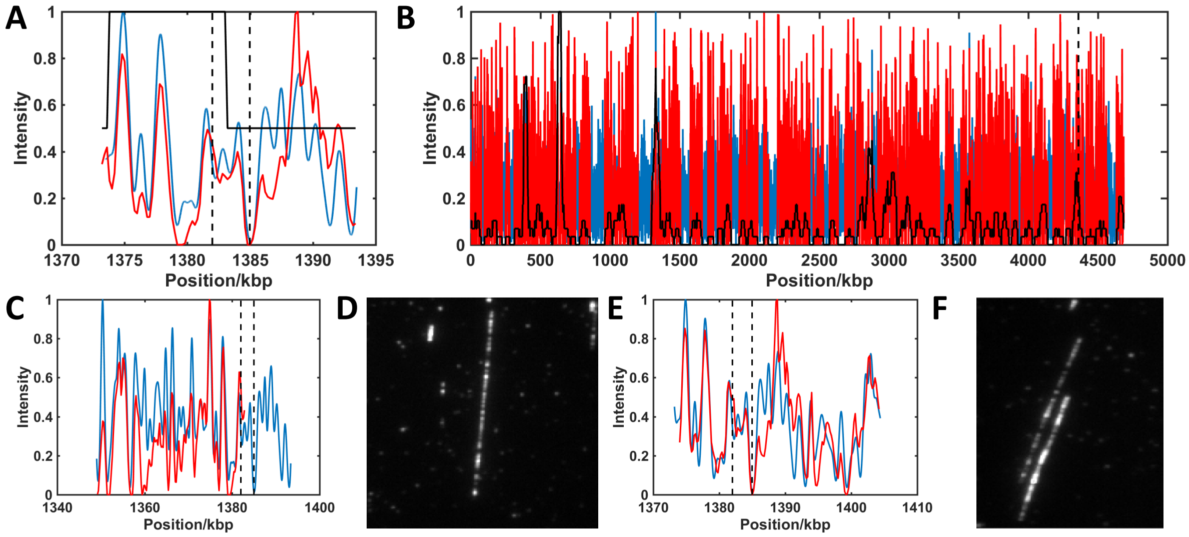


*Figure S9: Localisation of barcodes containing E.coli lacZ gene. Red lines show experimental barcode profiles. Blue lines show reference genome profiles. Black dashed lines show the expected position of the lacZ gene on the genome. Values in parenthesis below show alignment weight to reference. A) Consensus barcode generated from all barcodes overlapping with at least 25% of the region of interest. A maximum (minimum) of 2 (1) barcodes (solid black line) contribute to the consensus (0.734).B) Consensus of all barcodes aligned to the genome reference. A maximum (minimum) of 29 (0) barcodes (solid black line) contribute to the consensus across the genome (0.571). C,E) Single molecule barcodes aligned to region of interest (0.791, 0.764). D,F) Raw images of barcodes (shown in C and E, respectively) identified as overlapping region of interest.*

*Table S2: List of bacterial genomes and their accession numbers used in the strain-typing exercise. For each genome, the number of barcodes from the experimental dataset that are aligned with highest weighting to the reference genome are shown.*

| **Species and accession number** | **Number of matches per sample** | | |
|---|---|---|---|
| | **K. pneumoniae** | **E. coli DH10B** | **E. coli EC958** |
| 1: NC 000117.1 Chlamydia trachomatis D/UW-3/CX | 9 | 7 | 6 |
| 2: NC 000853.1 Thermotoga maritima MSB8 | 27 | 16 | 10 |
| 3: NC 000907.1 Haemophilus influenzae Rd KW20 | 8 | 2 | 7 |
| 4: NC 000911.1 Synechocystis sp. PCC 6803 DNA | 26 | 11 | 28 |
| 5: NC 000912.1 Mycoplasma pneumoniae M129 | 12 | 7 | 6 |
| 6: NC 000913.3 Escherichia coli str. K-12 substr. MG1655 | 47 | 68 | 48 |
| 7: NC 000915.1 Helicobacter pylori 26695 | 18 | 1 | 3 |
| 8: NC 000918.1 Aquifex aeolicus VF5 | 20 | 8 | 5 |
| 9: NC 000922.1 Chlamydophila pneumoniae CWL029 | 16 | 4 | 11 |
| 10: NC 000962.3 Mycobacterium tuberculosis H37Rv | 47 | 34 | 20 |
| 11: NC 000963.1 Rickettsia prowazekii str. Madrid E | 0 | 1 | 5 |
| 12: NC 000964.3 Bacillus subtilis subsp. subtilis str. 168 | 23 | 13 | 35 |
| 13: NC 001263.1 Deinococcus radiodurans R1 1 | 51 | 33 | 32 |
| 14: NC 001264.1 Deinococcus radiodurans R1 2 | 10 | 4 | 4 |
| 15: NC 001318.1 Borrelia burgdorferi B31 | 3 | 0 | 1 |
| 16: NC 002505.1 Vibrio cholerae O1 biovar El Tor str. N16961 I | 30 | 17 | 24 |
| 17: NC 002506.1 Vibrio cholerae O1 biovar El Tor str. N16961 II | 7 | 4 | 2 |
| 18: NC 002516.2 Pseudomonas aeruginosa PAO1 | 58 | 32 | 26 |
| 19: NC 002745.2 Staphylococcus aureus subsp. aureus N315 DNA | 37 | 12 | 19 |
| 20: NC 003098.1 Streptococcus pneumoniae R6 | 22 | 7 | 15 |
| 21: NC 003112.2 Neisseria meningitidis MC58 | 23 | 13 | 27 |
| 22: NC 003198.1 Salmonella enterica subsp. enterica serovar Typhi str. CT18 | 52 | 29 | 38 |
| 23: NC 003888.3 Streptomyces coelicolor A3(2) | 71 | 48 | 41 |
| 24: NC 011374.1 Ureaplasma urealyticum serovar 10 str. ATCC 33699 | 3 | 3 | 1 |
| 25: NC 021490.2 Treponema pallidum subsp. pallidum str. Nichols | 12 | 5 | 8 |
| 26: CP009114.1 Klebsiella pneumoniae strain blaNDM-1 | 79 | 31 | 42 |

*Table S3: List of E. coli strains and their accession numbers used in the strain-typing exercise. For each genome, the number of barcodes from the experimental dataset that are aligned with highest weighting to the reference genome are shown.*

| **Strain and accesion number** | **Number of matches per sample** | |
|---|---|---|
| | **E. coli DH10B** | **E. coli EC958** |
| 1: NC 004431.1 Escherichia coli CFT073 | 22 | 31 |
| 2: NC 008253.1 Escherichia coli 536 | 21 | 31 |
| 3: NC 010473.1 Escherichia coli str. K12 substr. DH10B | 52 | 25 |
| 4: NC 011415.1 Escherichia coli SE11 DNA | 27 | 16 |
| 5: NC 011750.1 Escherichia coli IAI39 chromosome | 20 | 18 |
| 6: NC 011751.1 Escherichia coli UMN026 | 26 | 20 |
| 7: NC 013654.1 Escherichia coli SE15 DNA | 20 | 46 |
| 8: NC 022648.1 Escherichia coli JJ1886 | 20 | 53 |
| 9: NZ CP008957.1 Escherichia coli O157:H7 str. EDL933 | 22 | 27 |
| 10: NZ HG941718.1 Escherichia coli ST131 strain EC958 chromosome | 21 | 56 |

*Table S4: Accession codes and quantitative data on the numbers of generated/aligned barcodes for a mixture of phage genomes, where barcodes were generated in silico for validating this approach.*

| Name | Length/bp | Accession Number | Generated | Found 1 | Found 2 | Found 3 | Found 5 | Found 5 | Min | Max | Mean |
|---|---|---|---|---|---|---|---|---|---|---|---|
| 'Stinger' | 69641 | NC_023741.1 | 430 | 489 | 552 | 522 | 511 | 503 | 489 | 552 | 515 |
| 'Babsiella' | 48420 | NC_023697.1 | 410 | 465 | 507 | 490 | 489 | 504 | 465 | 507 | 491 |
| 'SkiPole' | 53137 | NC_023748.1 | 390 | 485 | 584 | 551 | 511 | 531 | 485 | 584 | 532 |
| 'phiEf11' | 42822 | NC_013696.1 | 370 | 406 | 392 | 395 | 408 | 397 | 392 | 408 | 400 |
| 'DS6A' | 60588 | NC_023744.1 | 350 | 448 | 511 | 482 | 472 | 518 | 448 | 518 | 486 |
| 'lambda' | 48502 | NC_001416.1 | 330 | 367 | 347 | 356 | 358 | 358 | 347 | 367 | 357 |
| 'Alma' | 53177 | NC_023716.1 | 310 | 314 | 346 | 348 | 332 | 330 | 314 | 348 | 334 |
| 'Bruns' | 53003 | NC_023687.1 | 290 | 355 | 272 | 284 | 328 | 304 | 272 | 355 | 309 |
| 'phiMAM1' | 157834 | NC_020083.1 | 270 | 297 | 267 | 279 | 262 | 276 | 262 | 297 | 276 |
| 'T7' | 39937 | NC_001604.1 | 250 | 293 | 270 | 266 | 260 | 277 | 260 | 293 | 273 |
| 'GUmbie' | 57387 | NC_023746.1 | 230 | 299 | 415 | 308 | 306 | 289 | 289 | 415 | 323 |
| 'BarrelRoll' | 59672 | NC_023747.1 | 210 | 279 | 363 | 270 | 286 | 276 | 270 | 363 | 295 |
| 'Davies' | 45798 | NC_022980.1 | 190 | 203 | 201 | 201 | 200 | 204 | 200 | 204 | 202 |
| 'Maynard' | 154701 | NC_022768.1 | 170 | 0 | 0 | 45 | 64 | 94 | 0 | 94 | 41 |
| 'Slash' | 80382 | NC_022774.1 | 150 | 132 | 139 | 165 | 145 | 141 | 132 | 165 | 144 |
| 'PM1' | 50861 | NC_020883.1 | 130 | 179 | 159 | 163 | 167 | 158 | 158 | 179 | 165 |
| 'DeadP' | 56461 | NC_023728.1 | 110 | 192 | 0 | 114 | 130 | 116 | 0 | 192 | 110 |
| 'Yep_phi' | 38616 | NC_023715.1 | 90 | 118 | 74 | 90 | 79 | 0 | 0 | 118 | 72 |
| 'Acadian' | 69864 | NC_023701.1 | 70 | 103 | 87 | 97 | 146 | 112 | 87 | 146 | 109 |
| 'KBNP1711' | 76184 | NC_023593.1 | 50 | 0 | 0 | 0 | 0 | 0 | 0 | 0 | 0 |
| 'Rejected' | NaN | | 1200* | 576 | 514 | 574 | 546 | 612 | 514 | 612 | 564 |
| 'Other'† | NaN | | 0 | 0 | 0 | 0 | 0 | 0 | 0 | 0 | 0 |

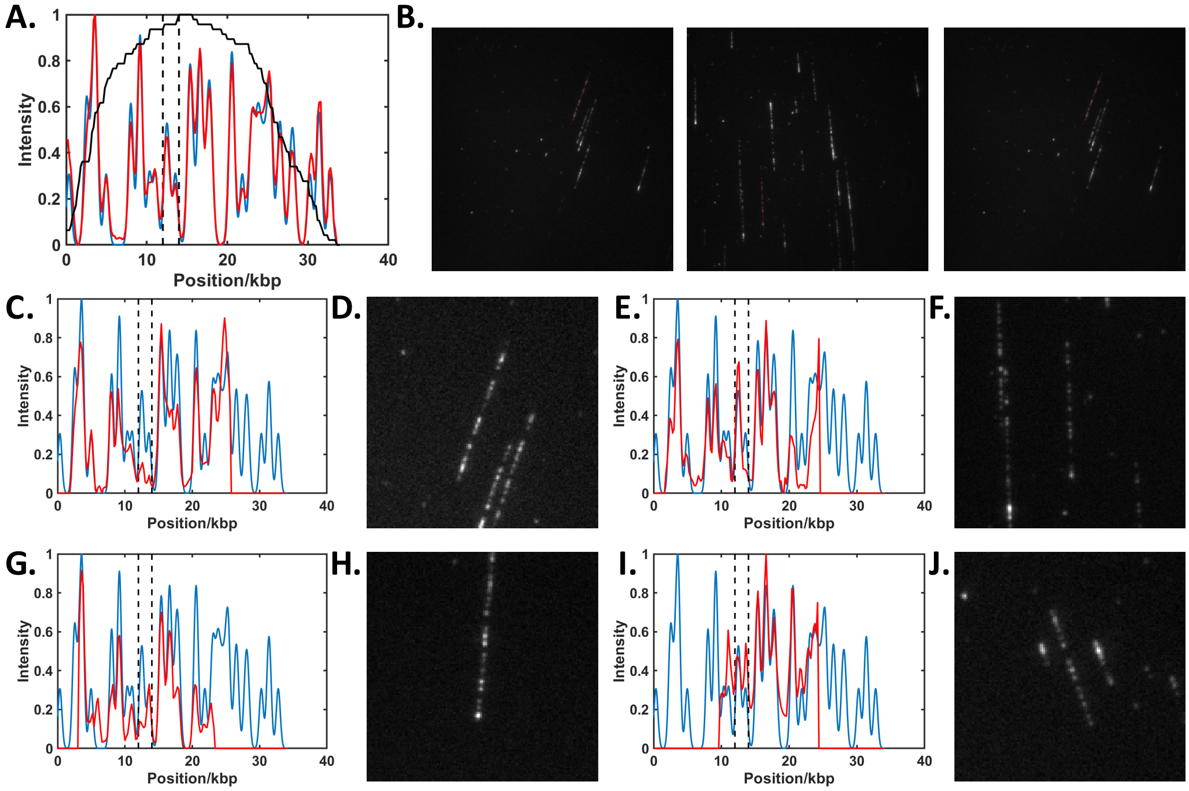


*Figure S10: Localisation of candidate barcodes of human adenovirus A (type 12) DNA against a background of DNA from host HeLa cells. Red lines show experimental barcode profiles. Blue lines show reference genome profiles. Black dashed lines show a region of interest on the genome. Values in parenthesis below show alignment weight to reference. A) Consensus of all barcodes aligned to the genome reference. A maximum (minimum) of 47 (0) barcodes (solid black line) contribute to the consensus across the genome (0.975). B) Typical full field of view images for the sample. Red dashed lines show the position of lines extracted in C, E, and G (left to right, respectively) C,E,G,I) Single molecule barcodes aligned to the region of interest (0.827, 0.825, 0.819, 0.809). D,F,H,J) Raw images of barcodes (shown in C,E,G, and I, respectively) identified as overlapping region of interest.*

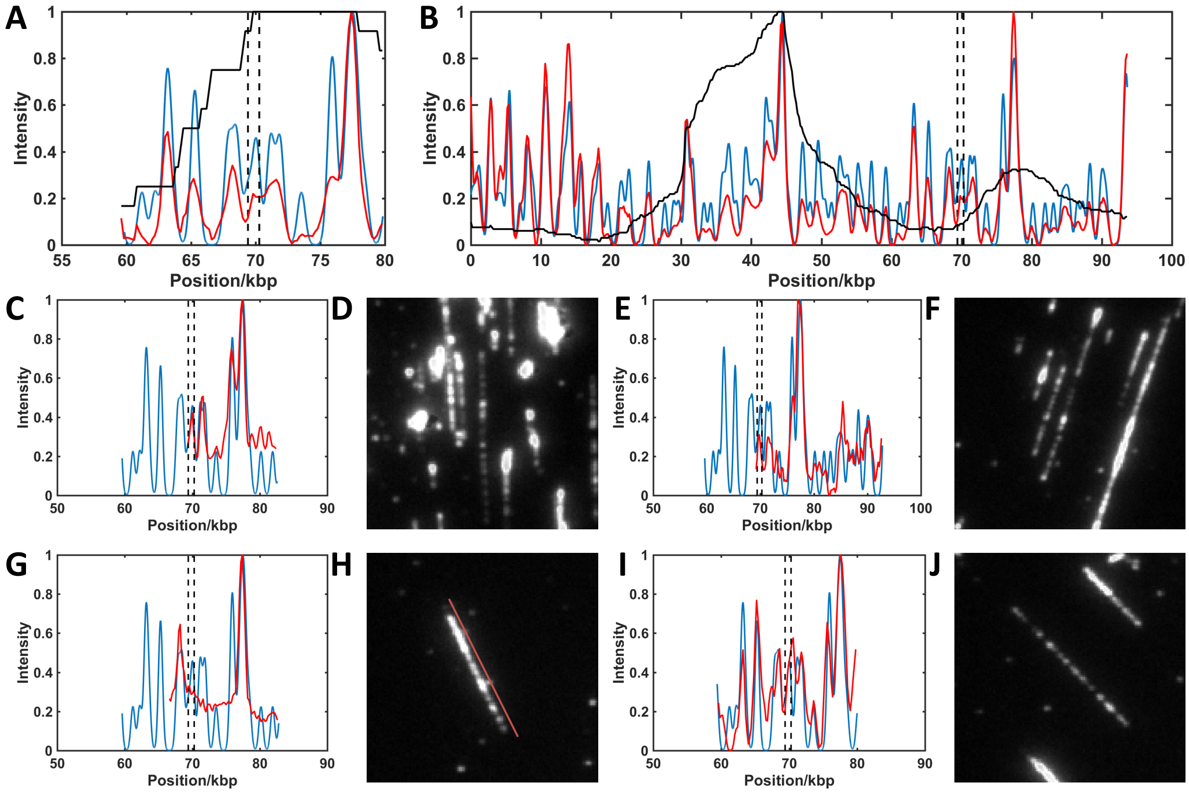


*Figure S11: Candidate barcodes for the blaCTX-M014b gene in an E. coli host. Red lines show experimental barcode profiles. Blue lines show reference plasmid (pCT, E. coli) profiles. Black dashed lines show the expected position of blaCTX-M014b in the plasmid. Values in parenthesis below show alignment weight to reference. A) Consensus barcode generated from all barcodes overlapping with at least 25% of the region of interest. A maximum (minimum) of 12 (2) barcodes (solid black line) contribute to the consensus (0.866).B) Consensus of all barcodes aligned to the plasmid reference. A maximum (minimum) of 133 (2) barcodes (solid black line) contribute to the consensus across the plasmid (0.866). C,E,G,I) Single molecule barcodes aligned to region of interest (0.808, 0.768, 0.764, 0.761). D,F,H,J) Raw images of barcodes (shown in C,E,G, and I, respectively) identified as overlapping region of interest. Note that the candidate barcode in G/H is likely an artefact, fitted to the reference with a high threshold due to the large peak in its profile.*

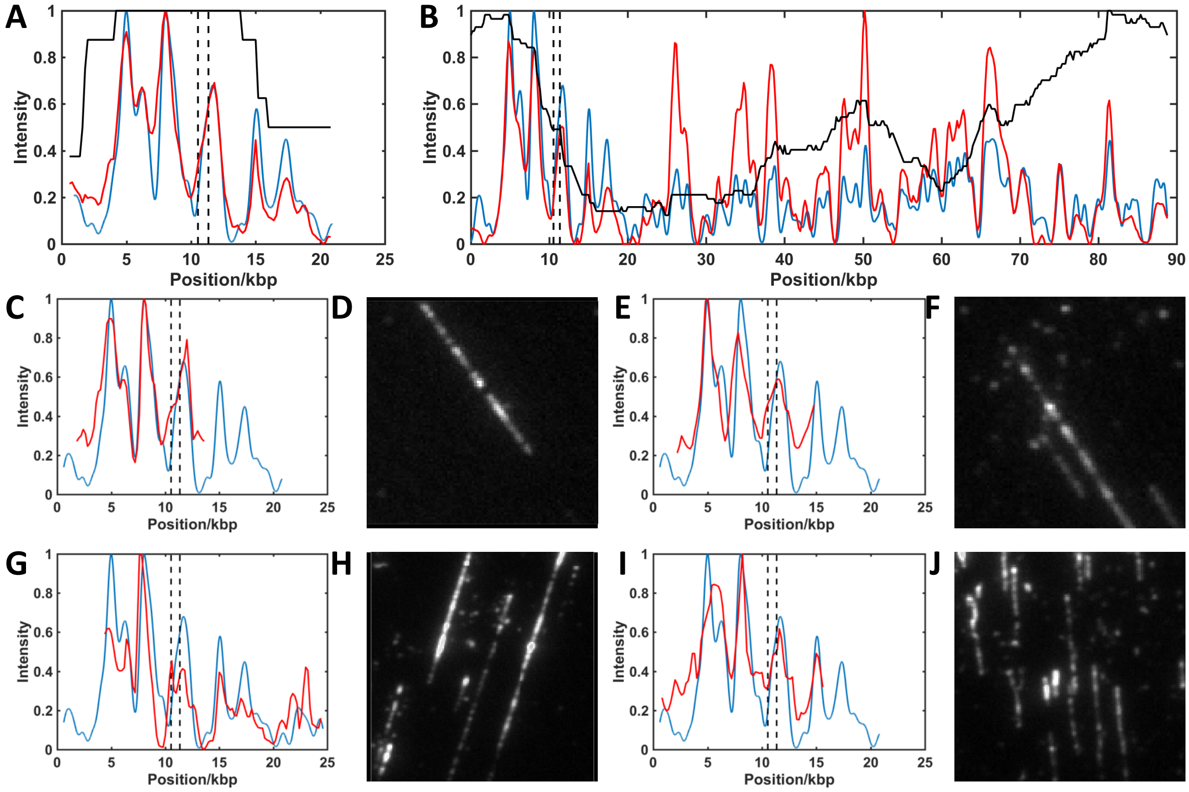


*Figure S12: Candidate barcodes for the blaNDM-1 gene in an E. coli host. Red lines show experimental barcode profiles. Blue lines show reference plasmid (pNDM, E. coli) profiles. Black dashed lines show the expected position of blaNDM-1 in the plasmid. Values in parenthesis below show alignment weight to reference. A) Consensus barcode generated from all barcodes overlapping with at least 25% of the region of interest. A maximum (minimum) of 8 (3) barcodes (solid black line) contribute to the consensus (0.918).B) Consensus of all barcodes aligned to the plasmid reference. A maximum (minimum) of 57 (7) barcodes (solid black line) contribute to the consensus across the plasmid (0.720). C,E,G,I) Single molecule barcodes aligned to region of interest (0.825, 0.795, 0.771, 0.767). D,F,H,J) Raw images of barcodes (shown in C,E,G, and I, respectively) identified as overlapping region of interest.*

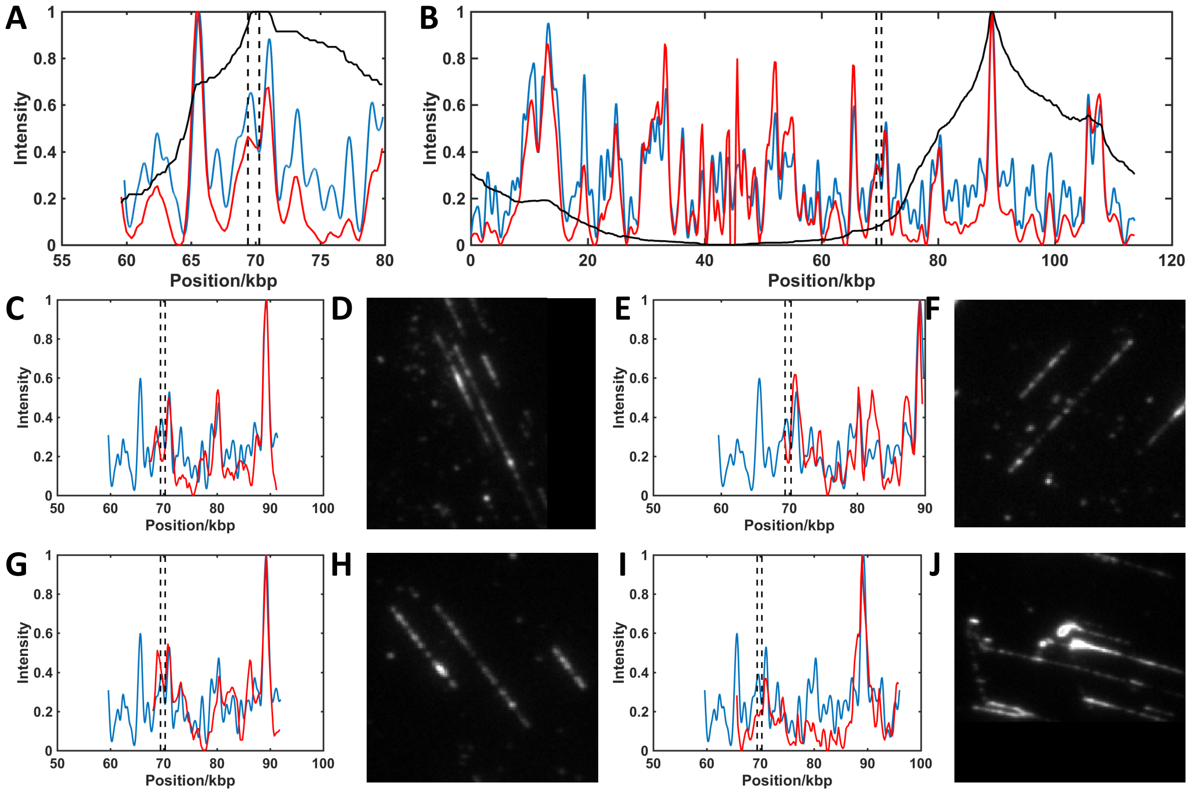


*Figure S13: Candidate barcodes for the blaCTX-M014b gene conferring antimicrobial resistance in a K. pneumoniae host. Red lines show experimental barcode profiles. Blue lines show reference plasmid (pKpQIL, K. Pne) profiles. Black dashed lines show the expected position of blaCTX-M014b in the plasmid. Values in parenthesis below show alignment weight to reference. A) Consensus barcode generated from all barcodes overlapping with at least 25% of the region of interest. A maximum (minimum) of 106 (19) barcodes (solid black line) contribute to the consensus (0.882).B) Consensus of all barcodes aligned to the plasmid reference. A maximum (minimum) of 1297 (0) barcodes (solid black line) contribute to the consensus across the plasmid (0.874). C,E,G,I) Single molecule barcodes aligned to region of interest (0.834, 0.810, 0.810, 0.810). D,F,H,J) Raw images of barcodes (shown in C,E,G, and I, respectively) identified as overlapping region of interest.*
